# Zebavidin - An Avidin-Like Protein from Zebrafish

**DOI:** 10.1371/journal.pone.0077207

**Published:** 2013-10-24

**Authors:** Barbara Taskinen, Joanna Zmurko, Markus Ojanen, Sampo Kukkurainen, Marimuthu Parthiban, Juha A. E. Määttä, Jenni Leppiniemi, Janne Jänis, Mataleena Parikka, Hannu Turpeinen, Mika Rämet, Marko Pesu, Mark S. Johnson, Markku S. Kulomaa, Tomi T. Airenne, Vesa P. Hytönen

**Affiliations:** 1 Institute of Biomedical Technology, University of Tampere, BioMediTech, Tampere, Finland; 2 Fimlab Laboratories, Pirkanmaa Hospital District, Tampere, Finland; 3 Institute of Molecular, Cell and Systems Biology, College of Medical, Veterinary and Life Sciences, University of Glasgow, Glasgow, United Kingdom; 4 Department of Biosciences, Biochemistry, Åbo Akademi University, Turku, Finland; 5 Tampere University Hospital, Tampere, Finland; 6 Department of Chemistry, University of Eastern Finland, Joensuu, Finland; 7 Department of Paediatrics, Tampere University Hospital, Tampere, Finland; University of Alabama at Birmingham, United States of America

## Abstract

The avidin protein family members are well known for their high affinity towards D-biotin and high structural stability. These properties make avidins valuable tools for a wide range of biotechnology applications. We have identified a new member of the avidin family in the zebrafish (*Danio rerio*) genome, hereafter called zebavidin. The protein is highly expressed in the gonads of both male and female zebrafish and in the gills of male fish, but our data suggest that zebavidin is not crucial for the developing embryo. Biophysical and structural characterisation of zebavidin revealed distinct properties not found in any previously characterised avidins. Gel filtration chromatography and native mass spectrometry suggest that the protein forms dimers in the absence of biotin at low ionic strength, but assembles into tetramers upon binding biotin. Ligand binding was analysed using radioactive and fluorescently labelled biotin and isothermal titration calorimetry. Moreover, the crystal structure of zebavidin in complex with biotin was solved at 2.4 Å resolution and unveiled unique ligand binding and subunit interface architectures; the atomic-level details support our physicochemical observations.

## Introduction

Chicken avidin [1] and its bacterial cousin streptavidin from *Streptomyces avidinii* [2] have been studied extensively for decades. Many biotechnological applications would be unthinkable without the presence of these stable proteins that have extremely high affinity (K_d_ = 10^-14^ - 10^-16^ M) towards biotin (D-biotin) [3].

 Avidin is found in the egg white of chicken and other egg-laying species [1]. Its expression in the oviduct is induced by progesterone, but it has also been shown to be induced in most tissues by bacterial infection or physical damage [4-6]. These findings led to the suggestion that avidin acts as an anti-microbial agent, yet its physiological function is still not completely understood.

 The sequencing of the zebrafish (*Danio rerio*) genome revealed a gene similar to that encoding chicken avidin [7]. Zebrafish, a tropical freshwater fish, is a model organism used traditionally to study developmental biology and more recently to investigate many other fields of biomedical research including haematological disorders and infectious diseases [8,9]. Although avidins have been found in many organisms including chickens and other birds, frog (xenavidin from *Xenopus tropicalis*, [10]), mushrooms (tamavidin from Tamogitake mushroom, [11]) and several bacterial species [2,12-14], no evidence for avidin proteins in fish species have been published to date. In an early study, Korpela et al. observed avidin expression in various vertebrates, but their studies did not include any fish species [15].

 In the current study, we present the characterisation of an avidin-like protein in zebrafish, called zebavidin. Expression of zebavidin and its effect on embryonic development in zebrafish was investigated. We used biochemical and biophysical methods to evaluate the ligand-binding properties, oligomeric state and thermal stability of the produced recombinant zebavidin. X-ray crystallography was used to analyse the structure at atomic detail. Although zebavidin has many unique physicochemical and structural features, it clearly can be classified as a new member of the avidin protein family based on its structure and function.

## Materials and Methods

### Zebrafish maintenance

Wild-type AB zebrafish were used in all experiments. The fish were maintained according to standard protocols [16,17]. The adult fish were kept in a flow-through system at 28 °C with a light/dark cycle of 14 h/10 h. The fish tanks were made of FDA-approved, good grade autoclavable polycarbonate USP class VI. Reverse osmosis water was used with conductivity (800 µS) and pH levels (pH 7.6) adjusted using sea salt (Instant Ocean, Blacksburg, VA, USA) and NaHCO_3_ respectively. The water was filtered mechanically using filters, chemically using activated carbon and sterilized with UV-light. Nitrifying bacteria were added to the system to convert nitrogenous wastes to less toxic substances. Water was partially (10%) exchanged daily. The fish were fed with SDS 400 food (Special Diets Services, Essex, UK) twice a day. Embryos were grown in E3-H_2_O (5mM NaCl, 0.17 mM KCl, CaCl_2_, 0.33 mM MgSO_4_, 1 · 10^-5^% methylene blue) at 28 °C. Fish were euthanized with 4 mg/ml Tricaine (ethyl 3-aminobenzoate methanesulfonate salt) pH 7; the pH was adjusted using Tris-buffer. All of the zebrafish experiments were in accordance with the Finnish Laboratory Animal Welfare Act 62/2006, the Laboratory Animal Welfare Ordinance 36/2006 and have been authorized (authorization LSLH-2007-7254/Ym-23) by the National Animal Experiment Board (Finland).

### Zebavidin gene expression analysis with qRT-PCR

Relative zebavidin mRNA quantification was performed with adult wild-type AB zebrafish tissue samples (gonads, gills, kidney, tail fin, eyes and brain) of both sexes as well as from whole developing wild-type AB strain embryos of different ages (0-7 days post fertilization (dpf)). Because of the difficulties of separating gametes from gonads, “gonad” samples contain tissue from both gonads and gametes. Developmental samples were pooled from 15-50 individual embryos depending on the age. Total RNA was extracted with an RNeasy RNA purification kit (Qiagen, Hilden, Germany) followed by reverse transcription using iScript™ Select cDNA synthesis kit (Bio-Rad, California, USA) according to the manufacturer’s instructions. Zebavidin primers (F: 5´-CGAATGCAAAGGTGAGCTCC-3´ and R: 5´-ATAGCACGGAGAAAGAGACG-3´) for quantitative real-time PCR (qRT-PCR) were ordered from Oligomer (Helsinki, Finland). The mRNA expression was measured using cDNA, qRT-PCR, Maxima SYBR Green qPCR master mix (Fermentas, Burlington, Canada) and a CFX96 qPCR machine (Bio-Rad Laboratories). Adult tissue samples and 0 dpf developmental samples were run as technical duplicates from 3-7 biological replicates and the 1-7 dpf developmental samples as technical triplicates from one pooled biological sample. Expression of zebavidin was normalized to elongation factor 1-alpha gene (*EF1a*, ENSDARG00000020850) expression [18]. Bio-Rad CFX Manager software (Bio-Rad) and Microsoft Office Excel 2010 were used in the analysis of the results. GraphPad Prism 5.0 (GraphPad Software, Inc., La Jolla, CA, USA) was used in the statistical analysis. Additionally, the qRT-PCR products were subjected to melting curve analysis followed by 1.5% agarose gel (Bioline, London, UK) electrophoresis.

### Morpholino studies

The zebavidin translation initiation site was sequenced from the genomic DNA of three zebavidin strains (+AB5, +AB7 and +AB8). The Morpholino, aimed at blocking the translation of zebavidin by binding to the translation initiation site (5’-GCCATATTAAAGAACTCATCTTGGC-3’), was ordered from GeneTools, LCC, (Philomath, USA). The Morpholino stock was diluted to a final concentration of 130 μM using 200 mM KCl containing 0.2% rhodamine dextran tracer (Invitrogen, Carlsbad, CA, USA). The rhodamine dextran tracer allowed the confirmation of a successful injection of the Morpholino using fluorescence microscopy. Morpholino solution, 1nl, was injected into the yolk of 150 one-cell stage zebrafish eggs. Ninety eggs were injected with a random control Morpholino with random sequence (5’-CCTCCTACCTCAGTTACAATTTATA-3’). The control Morpholino served as a negative control. Embryos were grown in E3-H_2_O at 28 °C and screened at 24 h post injection using a fluorescent microscope in order to determine the success of the injection. Developing embryos were analysed under a contrast light microscope after 24 and 48 h.

### Isolation of zebavidin from zebrafish oocytes

Zebrafish oocytes were extracted from zebrafish gonads of five individuals. The oocytes (20- 150 mg per individual) were washed with 1 ml PBS and centrifuged for 5 min at 5,000 g and 4 °C. Supernatant was removed, mixed with 20 µl biotin Sepharose™ 4 Fast flow (Affiland S.A., Ans Liege, Belgium) and incubated at 4 °C for 1 hour. Washed oocytes were resuspended in 400 µl 0.5 M sucrose, 0.01 mg/ml lysozyme, 1 mM EDTA, 200 mM Tris, pH 7.4 and incubated on ice for 30 min. 400 µl 2 mM EDTA, 150 mM NaCl, 1% TritonX-100, 50 mM Tris, pH 8 was added and oocytes were sonicated at 25% amplitude two times for 15 s (alternating 1 s on, 1 s off). Sonicated oocytes were centrifuged at 13,000 g and 4 °C for 20 min. PBS + 1 M NaCl (800 µl) and 20 µl biotin Sepharose™ were added and samples were incubated at 4 °C for 1 hour. Sepharose was collected by centrifugation at 2,500 g and 4 °C for 5 min. Sepharose was washed with 1 ml PBS + 1 M NaCl. Samples from each step were taken and analysed on 15% SDS PAGE and subsequent Coomassie Brilliant Blue staining. A molecular marker (Page Ruler™ Prestained Protein Ladder, Fermentas) and bacterial expressed zebavidin were used for the size determination. Four selected bands with a molecular weight corresponding to zebavidin (13 kDa) were excised from the gel and dried in acetonitrile. Samples were in-gel digested at the Proteomics Facility (Turku, Finland) according to the standard protocol and analyzed by liquid chromatography tandem mass spectrometry (LC-MS/MS) using the LTQ Orbitrap Velos Pro mass spectrometer. The obtained data was searched against the NCBInr database (release 2013_02) using Mascot 2.4.0 (Matrix Science, Boston, MA, USA). 

### Construction of expression vector

Zebavidin cDNA (GenBank: BC127392.1) was amplified from the cDNA obtained from the I.M.A.G.E cDNA collection (IMAGE:5413136) using the primers Zeb_pET101_5' (5’-CACCATGAGTTCTTTAATATGGCATTTGG-3’) and zeb_3'_HindIII (5’-TCAAGCTTAATTTGAAACTCCAGTCTTG-3’) for amplification of the whole cDNA including the natural secretion signal peptide. The sequence encoding for the OmpA secretion signal peptide from *Bordetella avium* was added to cDNA encoding the core region of zebavidin in a two-step stepwise elongation of sequence PCR (SES-PCR) process [19], essentially as described earlier for chicken avidin [20]. Primers zeb_OmpA_B.a_5’ (5’-GCCGCCGTTACGGCCTCTGGTGTTGCCTCGGCTCAGACCGTGAGCTCCTGTAATGTGACC-3’) and zeb_3'_HindIII were used in the first step and primers chim_to_2_5' (5’- CACCATGAACAAACCCTCCAAATTCGCTCTGGCGCTTGCCTTCGCCGCCGTTACGGCCTC-3’) and zeb_3’_HindIII in the second step of SES-PCR. The PCR intermediates and products were purified from the gel with an Illustra™ GFX™ PCR DNA and Gel Band Purification Kit (GE Healthcare, Buckinghamshire, UK) and subsequently cloned into the pET101/D plasmid (Invitrogen) using the TOPO^®^ cloning protocol followed by a standard heat shock transformation of chemically competent Top10 *E. coli* cells (Invitrogen). The sequence of the construct obtained by this method was confirmed using the BigDye® Terminator v3.1 Cycle Sequencing Kit (Applied Biosystems, Carlsbad, CA, USA).

### Recombinant protein production

Zebavidin was produced in a pilot scale fermentor as previously described [21]. The pET101/D vector containing the zebavidin ORF was transformed into *E. coli* BL21-AI (Invitrogen) cells. Single colonies were grown overnight in 5 ml of fermenting medium containing 100 μg/ml ampicillin and 10 μg/ml tetracycline at 27 °C with shaking at 200 rpm. The cell culture was diluted in 500 ml of fermenting medium (see 21) under the same conditions and used the following day to start a 4.5 l fermentation in a Labfors Infors 3 fermentor (Infors HT, Bottmingen, Switzerland) at 25 °C. The fermenting medium contained the antifoam agent struktol J 647 (Schill + Seilacher, Hamburg, Germany). The culture was induced with 1 mM isopropyl β-D-1-thiogalactopyranoside (IPTG) and 0.2% (w/v) L(+)-arabinose when the OD_600_ reached a value of approximately 20. The target pO_2_ of the culture was set to 20%, which was controlled by the agitation speed (200-1150 rpm) and air flow rate. The feeding of the culture was controlled by a pO_2_-stat, which applies feed when the oxygen level rises above 40%. In practice, this was achieved by allowing the pO_2_ to oscillate during the feeding phase. Fermentation was stopped 24 h after inoculation.

### Zebavidin purification by affinity chromatography

The cell pellet was suspended in PBS buffer containing 2 mM EDTA and 1 M NaCl (for biotin affinity chromatography), or 50 mM Na-carbonate (pH 11) containing 1 M NaCl (for 2-iminobiotin affinity chromatography). The lysate was homogenised twice using an EmulsiFlex C3 homogeniser (Avestin Inc., Ottawa, Canada) using guiding pressure set to 40 psi to obtain homogenizing pressure of 15,000 psi. The crude cell lysate was clarified by centrifugation at 16,000 g and 4 °C for 60 min. The protein was purified by affinity chromatography as described by K. Airenne et al. [22] using biotin or 2-iminobiotin Sepharose™ 4 Fast flow (Affiland S.A., Ans Liege, Belgium). The protein concentrations in the eluted fractions were determined using the theoretical molar absorption coefficient of zebavidin at 280 nm (28,990 M^-1^cm^-1^) and the estimated molecular weight (13,588.70 g/mol). The A_280_ values were measured using a NanoDrop 2000 (Thermo Scientific, Wilmington, DE, USA). The molecular size and purity of the protein were analysed by SDS-PAGE (15% gel) and subsequent Coomassie Brilliant Blue staining. A molecular marker (Page Ruler™ Prestained Protein Ladder, Fermentas) was used for the size determination.

### Analytical gel filtration

The molecular size of the protein in solution was measured by size-exclusion chromatography using a Superdex200 10/300GL column connected to an ÄKTA™ purifier-100 equipped with a UV-900 monitor (GE healthcare/Amersham Biosciences AB, Uppsala, Sweden). As a mobile phase, either 50 mM sodium phosphate (NaH_2_PO_4_/Na_2_HPO_4_), 2 mM EDTA, pH 7 or 10 mM ammonium acetate, 0.05% β-mercaptoethanol, pH 7 with varying concentrations of sodium chloride (0, 100 and 650 mM) was used. Approximately 40-80 μg of protein was injected per run. All analyses used a flow rate of 0.3 ml/min and were executed at 4 °C. Absorbance at 280 nm was used to detect the eluting protein. A molecular weight calibration curve was prepared for each buffer condition by using a gel filtration standard protein mixture containing thyroglobulin (670 kDa), γ-globulin (158 kDa), ovalbumin (44 kDa) and myoglobin (17 kDa) (Bio-Rad).

### Mass spectrometry

Mass spectrometric (MS) analyses for the characterization of structural and functional properties of recombinant zebavidin were performed on a 12-T APEX-Qe™ Fourier transform ion cyclotron resonance (FT-ICR) instrument (Bruker Daltonics, Billerica, MA, USA), interfaced to an electrospray ionisation (ESI) source. The protein sample in 2 M acetic acid was buffer exchanged into 10 mM ammonium acetate (pH 3.0) with the use of PD-10 columns (GE Healthcare, Uppsala, Sweden). The resulting fractions, which eluted between 4 and 6 ml, were pooled and concentrated to approximately 250 μl using Millipore Ultrafree-0.5 Biomax-5 (5-kDa cut-off) centrifugal filter devices (Millipore, Billerica, MA, USA). The concentrations of the protein stock solutions were determined as described above. In order to analyse the protein under denaturing solution conditions, the stock solution was further diluted with an acetonitrile/water/acetic acid (49.5:49.5:1.0, v/v) solvent. Alternatively, the sample was diluted with 10–500 mM ammonium acetate (pH ~7) to perform native-MS analysis. ESI-generated ions were externally accumulated for 1 s in the hexapole ion trap before being transmitted to the ICR cell for trapping, excitation and detection. For each spectrum, a total of 250 co-added 1MWord (128kWord for native-MS) time-domain transients were zero-filled once prior to a fast Fourier transformation, magnitude calculation and external mass calibration with respect to the ions of an ES Tuning Mix (Agilent Technologies, Santa Clara, CA, USA). The instrument was operated and the data were processed with the use of Bruker XMASS 6.0.2 software.

### Fluorescence spectroscopy

The affinity of the zebavidin protein towards the biotin-labelled fluorescent dye ArcDia™ BF560 (ArcDia, Turku, Finland) was measured by a method based on the quenching of the dye as a result of its binding to avidin [20]. In practice, a 10 nM solution of the BF560 probe in 50 mM sodium phosphate, 650 mM NaCl and 0.1 mg/ml BSA was titrated with a known concentration of the protein at 25 °C with continuous stirring. The sample was allowed to equilibrate for 10 min after each addition of protein, and the fluorescence intensity at 578 nm was measured for 20 s following excitation at 560 nm. The protein was added to the sample until no clear decrease in fluorescence intensity was observed, indicating the point where nearly all available ligand-binding sites were occupied by biotin. The concentration of the protein where half of the available ligand-binding sites are occupied corresponds to the equilibrium dissociation constant (K_d_), which was calculated by fitting a binding curve to the data by nonlinear regression using Origin 7.0 (Originlab Corporation, Northampton, MA, USA). 

 The dissociation rate constant (k_diss_) of fluorescently labelled biotin was determined by fluorescence spectrometry using the biotin-labelled fluorescent probe ArcDia™ BF560 as described in [20]. In principle, the changes in the fluorescence intensity of a 50 nM dye in a pH 7 buffer (50 mM sodium phosphate, 650 mM NaCl, 0.1 mg/ml BSA) were measured after the addition of 100 nM biotin-binding protein. The dissociation of this complex was observed by addition of a 100-fold molar excess of free biotin (D-biotin, Sigma-Aldrich Co. LLC., St. Louis, MO, USA). The assay was performed at 25 °C and 50 °C using a QuantaMaster™ Spectrofluorometer (Photon Technology International, Inc., Lawrenceville, NJ, USA).

### Radioactive [^3^H]biotin assay

The biotin-binding properties were further studied with a radioactive biotin assay modified from that described in [23]. The protein at 50 nM subunit concentration was incubated with 10 nM radioactive biotin ([8,9-^3^H]biotin, PerkinElmer, Waltham, MA, USA) at room temperature (22 ± 1 °C) for 20 min. Measurements were performed in 50 mM NaH_2_PO_4_/Na_2_HPO_4_, pH 7, containing 100 mM NaCl and 10 µg/ml BSA to prevent non-specific binding. Centrifugal ultrafiltration was performed through 30,000 MW cut-off filter (Vivaspin 500 centrifugal concentrators, Sigma-Aldrich) to separate the unbound [^3^H]biotin from the protein-ligand complex. Excess cold biotin (D-biotin, Sigma-Aldrich) was added to a final concentration of 50 µM to replace [^3^H]biotin and measure its dissociation. The dissociated [^3^H]biotin was separated from the protein-ligand complex at different time points by ultrafiltration and the radioactivity of the filtrate was analysed in a Wallac 1410 liquid scintillation counter (Wallac Oy, Turku, Finland). Triplicates of each sample were measured at each time point.

Equation (1) was fitted to the data in order to determine the fraction of bound radioactive biotin at each time point:

$$-k_{diss}t=\ln\left[\frac{\left(x_{t}-x\right)}{\left(x_{t}-x_{0}\right)}\right]=\ln(fraction\_bound)$$ (1)

where *x*_*t*_ is the total amount of radioactive biotin before the addition of protein, *x* is the free biotin at each time point and *x*_*0*_ is the amount of free ligand in the presence of protein immediately prior to the addition of cold biotin. The dissociation rate constant (k_diss_) was determined from the slope of the linear fit to the data points of ln(fraction bound) versus time [23].

### Isothermal Titration Calorimetry (ITC)

Affinity towards biotin was determined using a high-sensitivity VP-ITC titration calorimetry instrument (Microcal Inc., Northampton, MA, USA) by using direct titration with biotin as described in [24] and by using a competitive titration method as described earlier in [25]. Measurements were made in 50 mM NaH_2_PO_4_/Na_2_HPO_4_, 100 mM NaCl, 2 mM EDTA, pH 7. Ligand solutions of biotin and desthiobiotin (Sigma-Aldrich) with a concentration of 75 µM were prepared in the same dialysis buffer as the protein. The protein solution in the cell was 4.65 µM. At first, a biotin titration experiment to zebavidin was carried out by using 15 µl aliquots. Competitive binding experiments were carried out as follows: In the first experiment, desthiobiotin was titrated in 15 µl aliquots to the zebavidin. In a second experiment, biotin was titrated in 15 µl aliquots to a mixture of 4.65 µM zebavidin and 14 µM desthiobiotin. Measurements were done at 40 °C. Origin 7.0 (Originlab Corporation) was used to derive the association binding constant (K_a_) and enthalpy of binding (ΔH) from the measured biotin or desthiobiotin binding data. The observed values for desthiobiotin were then used to analyse the competitive titration reaction utilizing the “competitive binding” tool. The Gibbs free energy of binding (ΔG) and entropy (ΔS) were calculated from K_a_ and ΔH using equation (2) and (3) where R is the gas constant and T the temperature:

$$\Delta G=-RT\ln K_{a}$$ (2)

ΔG=ΔH−TΔS (3)

### Differential Scanning Calorimetry (DSC)

The thermal stability of the studied proteins in the presence and absence of ligands was analysed using an automated VP-Capillary DSC System (Microcal Inc.). Thermograms were recorded between 20 and 140 °C with a heating rate of 120 °C/h. Proteins were dialysed into 50 mM NaH_2_PO_4_/Na_2_HPO_4_, 2 mM EDTA, pH 7 or 10 mM ammonium acetate, 0.05% β-mercaptoethanol, pH 7. Samples with different salt concentrations were prepared by adding 5 M NaCl. Samples were degassed prior to the measurement. The protein concentration in the cell was 20-30 μM, and the ligand concentration was 60-90 μM. The results were analysed using the Origin 7.0 DSC software suite (Originlab Corporation).

### Stability analysis

The thermostability and oligomeric state of zebavidin in the presence and absence of biotin was measured using the SDS-PAGE stability assay as described previously [26,27]. In brief, zebavidin was chemically acetylated, SDS-PAGE sample buffer containing SDS and 2-mercaptoethanol was added, and the sample was heated to the target temperature for 20 minutes. The oligomeric state after treatment was then assayed by SDS-PAGE.

### Crystallization and X-ray structure determination

Zebavidin (1.6 mg/ml; 50 mM Tris-HCl, pH 7) was crystallized using the vapour diffusion method, 96-well sitting drop iQ plates (TTP Laptech), TTP Labtech’s mosquito^®^ liquid handling robot and a cooled temperature-controlled crystallization incubator (RUMED**^®^** model 3201) set-up for 22 °C. The protein was mixed with biotin solution (1 mg/ml; 5 mM Tris, pH 8.8, 8 mM CHES, pH 9.5) in 10:1 v/v ratio, respectively, before crystallization. The initial hit was found from the JCSG-plus^TM^ Screen (Molecular Dimensions) and, after optimization, 300 nl of the protein-biotin solution and 150 nl of well solution (0.18 M magnesium chloride, 0.09 M Bis-Tris, pH 5.5, 23% w/v PEG 3350) were used to crystallize zebavidin. The crystals formed typically in 1-2 weeks and their X-ray diffraction properties were initially analyzed directly from drops on 96-well plates using a PX Scanner (Agilent Technologies).

 X-ray data were collected at the ESRF beam line ID23-2 (Grenoble, France) at 100 K from a single crystal. As a cryoprotectant, 1 μl of glycerol (30% v/v in well solution) was added to the crystallization drop just prior to freezing in liquid nitrogen. The data were processed with XDS [28] (see Table 1 for statistics) and initial phase estimates for the structure factors were obtained using the molecular replacement program Phaser [29] within the CCP4i GUI [30,31]. For molecular replacement, a tetrameric homology model of zebavidin was created using Modeller [32] of Discovery Studio 3 (Accelrys Software Inc.) and AVR2 [PDB: 1WBI] [33] as the template structure; the zebavidin sequence was aligned with the sequences of chains A-D of the AVR2 structure using the program Malign [34] of the Bodil multi-platform software package for biomolecular visualization and modeling [35]. In Phaser, a solution could be found by searching four poly(Ala/Gly) models with trimmed loops and termini. The initial X-ray structure of zebavidin was refined with Refmac5 [36] and manually edited/rebuilt using Coot [37]. Solvent atoms, biotin molecules and a glycerol molecule were added to the model either with the automatic procedure of Coot and ARP/wARP [38-40], or manually in Coot. For structure determination statistics, see Table 1.

**Table 1:** X-ray structure determination statistics for zebavidin [PDB: 4BJ8].

**Cell parameters**	
Space group	*P*2_1_2_1_2
Unit cell:	
a, b, c (Å)	182.2, 196.8, 52.6
α, β, γ (°)	90, 90, 90
**Data collection^a^**	
Wavelength (Å)	0.87260
Beamline	ID23.2 (ESRF)
Detector	MarCCD
Resolution (Å)^b^	25 - 2.4 (2.5 - 2.4)
Unique observations^b^	74935 (8359)
I/sigma^b^	14.4 (3.4)
*R*_factor_ (%)^b^	12.0 (60.8)
Completeness^b^	100 (99)
Redundancy^b^	8.2 (8.3)
**Refinement**	
*R*_work_ (%)^c^	19.2
*R*_free_ (%)^c^	26.6
Monomers (asymmetric unit)	16
Protein atoms	14505
Heterogen atoms	262
Solvent atoms	503
*R.m.s.d:*	
Bond lengths (Å)	0.013
Bond angles (°)	1.7

a The numbers in parenthesis refer to the highest resolution bin.

b From XDS [28].

c From Refmac 5 [36].

 The final structure of zebavidin was validated using the inbuilt tools of Coot [37], and using MolProbity [41] of the Phenix software suite [42], before deposition to the Protein Data Bank [43,44] with PDB entry code 4BJ8.

### Miscellaneous methods

Primer design and sequence analysis were performed using DNAMAN 4.11 (Lynnon Corporation, Quebec, Canada). Signal peptide profiling was performed with SignalP [45,46]. N-glycosylation sites were predicted using NetNGlyc 1.0 Server [47]. The structural superimpositions were made using the “align” command of PyMOL (The PyMOL Molecular Graphics System, Version 1.5.0.2, Schrödinger, LLC) and the chain A (or tetramer ABCD) of zebavidin as the reference structure. The structure-based sequence alignment was done using Malign [34] in the visualization and modelling package Bodil [35]. The diagram showing hydrogen bonds to biotin was made using LigPlot^+^ v.1.4 [48]. ESPript [49] was used for visualization of the sequence alignment. PyMOL was used to create all of the figures relating to structural representations (except the diagram describing the hydrogen bonds). GIMP 2.6.9, CorelDRAW X5 and Photoshop CS5.1 were used to edit the figures.

## Results

### Analysis of an avidin-related gene in zebrafish

**Figure 1:**
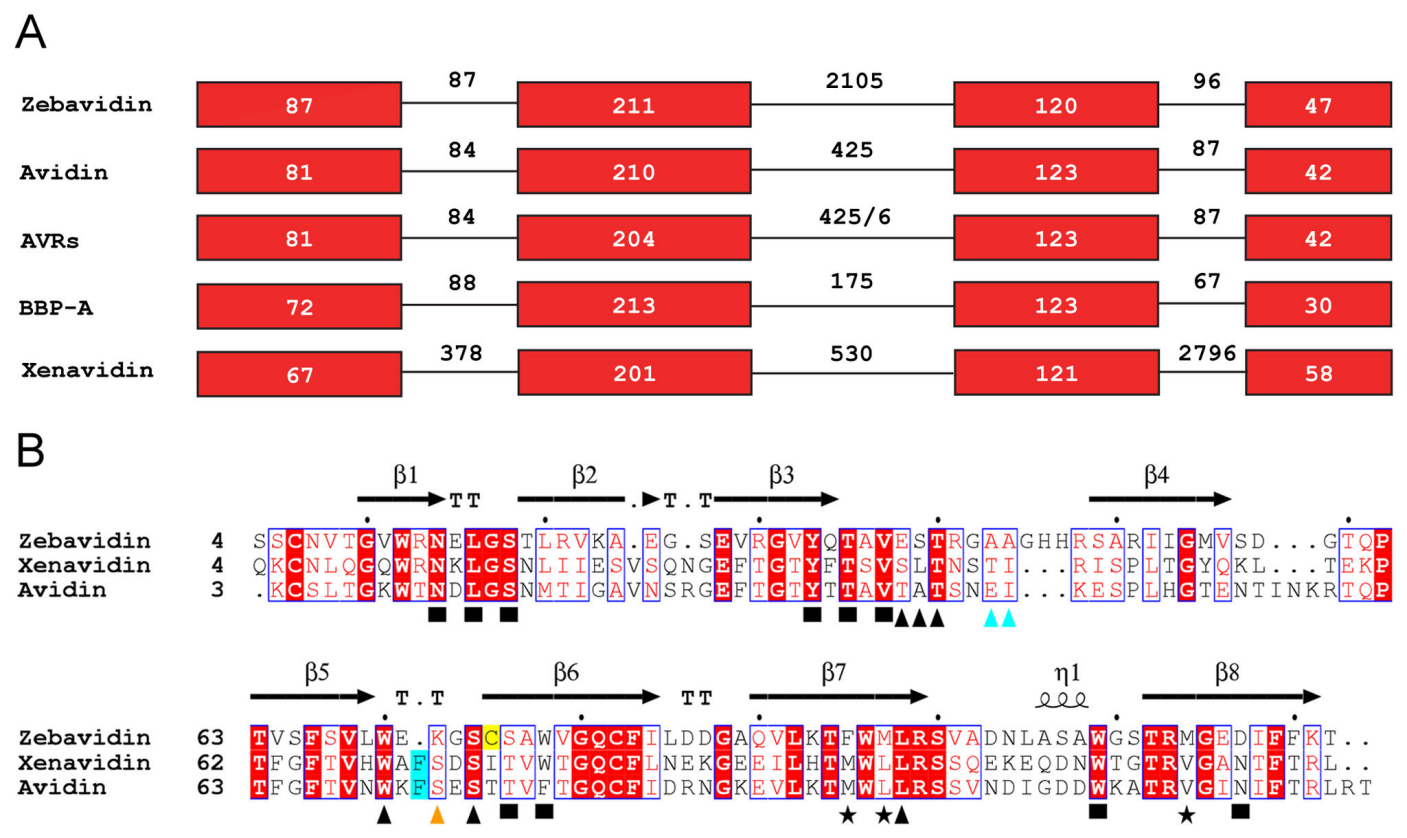
Gene organisation and structure-based sequence alignment of selected members of the avidin protein family. (A) Gene organization shown for selected avidins. The exons are shown as red boxes and the length of introns and exons are indicated. (B) Structure-based sequence alignment. The sequences of zebavidin [PDB: 4BJ8; reported here], xenavidin [PDB: 2UYW] and chicken avidin [PDB: 1AVD] are shown. Black squares: residues surrounding the bicyclic ring system of biotin; black triangles: residues around the valeric acid moiety; cyan triangles: zebavidin-specific Ala43 and Ala44 facing the valeric acid moiety of biotin; cyan background: the conserved Phe72/Phe71 of avidin/xenavidin missing from zebavidin; orange triangle: indicates the residue directly involved in biotin binding in avidin/xenavidin (Ser73/Ser72) but not in zebavidin (Lys72); asterisks: three zebavidin specific residues (Phe95, Met97 and Met114) found at IF1,2 and IF1,3 subunit interfaces; yellow background: the free cysteine (Cys75) of zebavidin. The conserved residues are indicated by the default coloring scheme of the ESPript program [49]. The β-strands of zebavidin are numbered. TT, β-turn and α1, 3/10-helix.

The DNA sequence from the genome assembly of zebrafish revealed a gene similar to the chicken avidin gene (Gene ID: 567678, Gene Symbol LOC567678). The gene is located on chromosome 10 of the *Danio rerio* genome, and it is 2981 base pairs in length in a region from base 18’857’183 to base 18’860’163. Alignment of zebavidin cDNA with its corresponding DNA sequence revealed that the zebavidin gene consists of four exons (87 bp, 211 bp, 120 bp and 42 bp) and three introns (87 bp, 2105 bp and 96 bp). This exon-intron structure is highly similar to that observed in all previously characterised avidins (Figure 1A). Unlike the chicken, which carries several avidin or avidin-related genes [50,51], zebrafish appears to have only one avidin-related gene. A protein sequence alignment of three eukaryotic avidins, zebavidin, xenavidin [10] and chicken avidin [52], shows that zebavidin shares many of the highly conserved residues involved in biotin binding but has also unique features (Figure 1B; see below).

### Zebavidin expression in zebrafish

**Figure 2:**
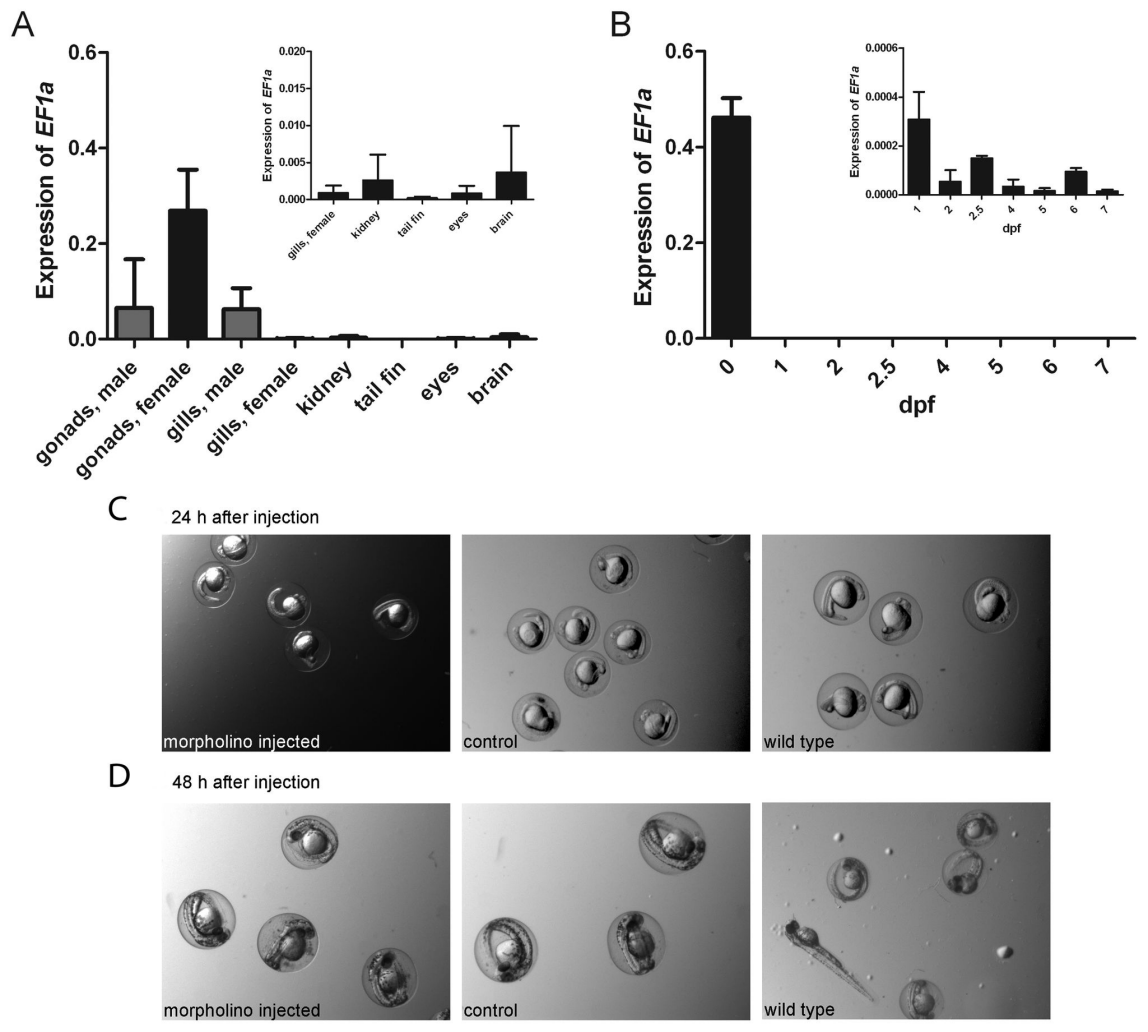
Expression of zebavidin in zebrafish. Relative zebavidin mRNA quantification was performed with qRT-PCR (A) in adult zebrafish tissues and (B) in developing zebrafish (0-7 days post fertilization (dpf)). Expression analysis of each tissue in (A) are shown as the mean (error bar = SD) of the biological replicates. The two-sample t-test was used to compare the expression between female gonads and female gills (P = 0.002), male gonads and male gills (nonsignificant, NS), female gonads and male gonads (P = 0.023) and female gills and male gills (NS). Expression analysis of the developmental samples in (B) are shown as the mean (SD) of the biological replicates on 0 dpf and as the mean (SD) of the technical replicates on 1-7 dpf. All of the developmental samples were pooled from 15 to 50 individuals depending on the age of embryos. The two-sample t-test was used to compare the expression of 0 dpf and 1 dpf samples to later time points. A statistically significant difference in expression was found between the 0 dpf sample and all of the other samples (P < 0.0001 in all comparisons) and also between the 1 dpf sample and the samples of 2, 4, 5, 6 and 7 dpf (P < 0.05 in all comparisons). In both (A) and (B) the expression of zebavidin is normalized to *EF1a* expression. The insets in the upper right corners represent enlargements of parts of the actual figures. (C) Embryos, injected with a Morpholino blocking the translation initiation site or with a random control Morpholino, 24 h after injection in comparison with WT embryos. (D) Embryos 48 h after injection.

In order to analyze the spatiotemporal expression of zebavidin, we quantified the expression of zebavidin with qRT-PCR in adult zebrafish and zebrafish embryos. The expression of zebavidin was normalized to the elongation factor 1-alpha gene (*EF1a*) housekeeping gene expression, and the relative zebavidin mRNA quantification was used to assess zebavidin mRNA amounts in male gonads (n=3), female gonads (n=5), male gills (n=3), female gills (n=3), kidney (n=6), tail fin (n=5), eyes (n=7) and brain (n=7). Zebavidin expression levels were also quantified in developing fish embryos of 0-7 days post fertilization (dpf, Figure 2 A&B).

 In adult zebrafish (see Figure 2A), the highest zebavidin expression was observed in female gonads. Expression of the zebavidin gene in ovaries was already observed to be higher as compared to the standard in a cDNA microarray [7]. Expression was also evident in male gonads and male gills. Interestingly, zebavidin mRNA was present only in very low amounts in female gills. Low concentrations of zebavidin mRNA were also present in kidney, tail fin, eyes and brain. Supporting these findings, the zebavidin protein was isolated from oocytes and its surrounding tissue and its identity was confirmed by MS analysis (Table S1 and Figure S1).

In the developmental samples (see Figure 2B), the relative zebavidin expression was observed to be the highest immediately after spawning at the 0 dpf timepoint. This expression was over a thousand fold higher than at the next timepoint of 1 dpf (P < 0.0001, two-sample t-test). This, together with the high expression levels in the maternal gonads, implies that zebavidin has been expressed in the mother prior to spawning and that the high level of zebavidin mRNA in the 0 dpf embryo is of maternal origin. This is further supported by the results from the Morpholino study: injection of a zebavidin-specific Morpholino, designed to block zebavidin protein production by binding to the translation initiation site caused no detectable effects during the 2-day follow-up (Figure 2C). However, the efficiency of the Morpholino was not confirmed by any other experiments.

### Expression of recombinant zebavidin in *E. coli*

The signal peptide prediction program SignalP [46,53] suggested two probable N-terminal signal peptide cleavage sites located between amino acid residues 21 and 22 and residues 29 and 30. According to this analysis with SignalP, a secretion signal was found that is potentially functional in gram-negative bacteria too. Therefore, the entire open reading frame of zebavidin cDNA was first constructed into the pET101/D expression vector. However, no active protein was expressed using this construct. Therefore, the first 30 amino acids of the open reading frame were replaced with an N-terminal secretion signal peptide OmpA from *Bordetella avium* [20,54]. The N-terminal truncation was based on the results from the SignalP analysis and on sequence comparison of known avidins. The residues analogous to those cleaved away from zebavidin are not known to have any important role in other avidins. Purification of *E. coli* expressed zebavidin was attempted with two different affinity chromatography columns; purification with 2-iminobiotin Sepharose^TM^ yielded an insignificant amount of the protein, whereas zebavidin bound well to the biotin column and could be eluted using a stepwise gradient of acetic acid (0-4 M) with typical yields of 175 µg of recombinant zebavidin per gram of cell pellet (wet weight).

### Identification of the *E. coli* expressed and purified protein

The identity of the purified protein was confirmed by ESI FT-ICR mass spectrometry analysis under denaturing solution conditions (Figure S2). The most abundant isotopic mass for the zebavidin monomer M (averaged over the detected protein ion charge states 8+ to 13+) was 13,588.80 ± 0.03 Da, which is in a good agreement with the theoretical mass (13,588.70 Da) calculated from the amino acid sequence. This analysis suggests the presence of one intramolecular disulphide bond, one free cysteine per monomer and no intermolecular disulphide bonds between subunits, all of which is in agreement with the X-ray structure.

### Dynamic oligomeric state of zebavidin

**Figure 3:**
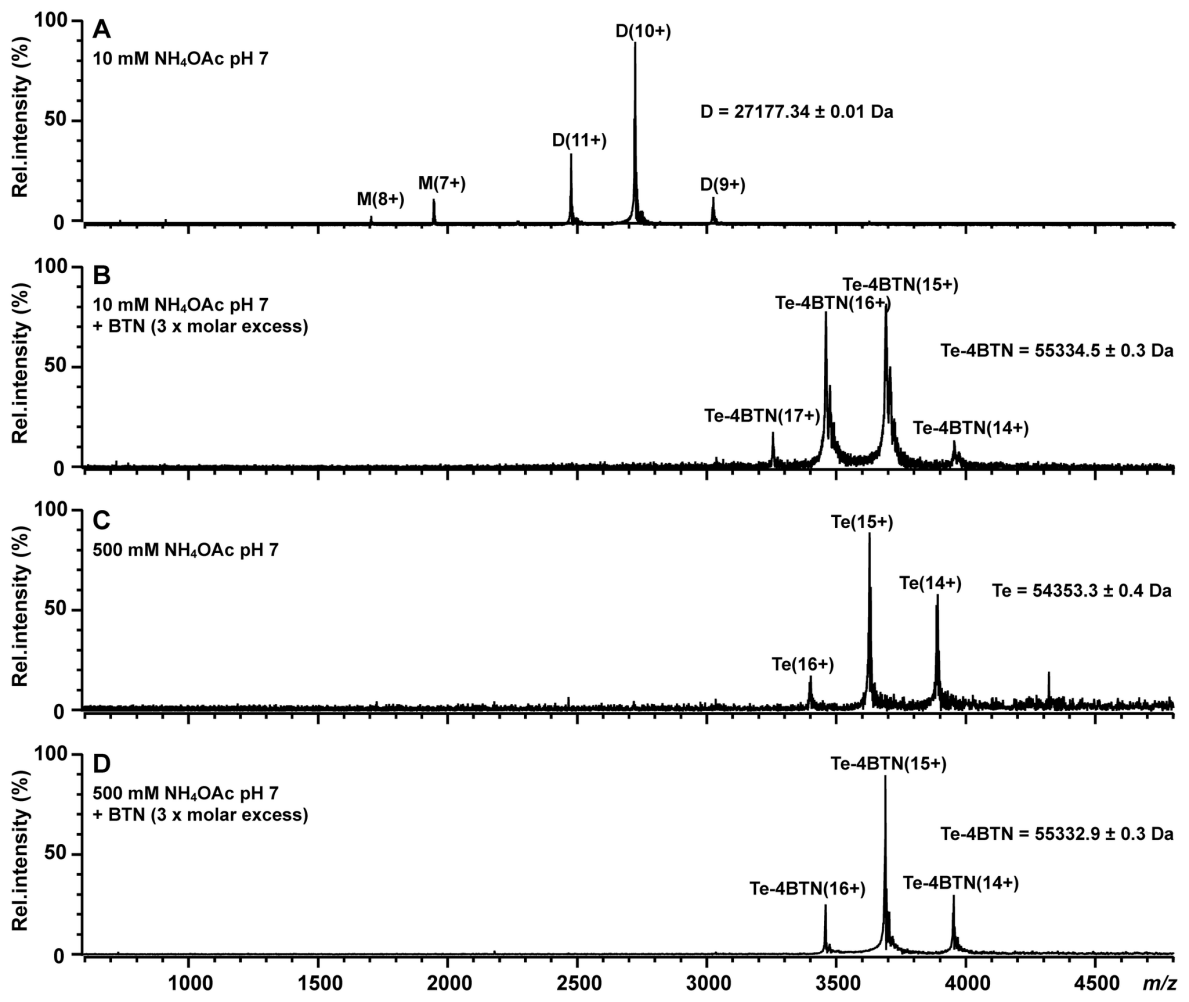
Native-MS analysis of zebavidin. Native mass spectra of 10 µM zebavidin in four native solution conditions: ammonium acetate buffer 10 mM (A), 10 mM + 30 µM biotin (BTN) (B), 500 mM (C) and 500 mM + 30 µM biotin (D). Letters M, D and Te correspond to the protein monomer, dimer and tetramer, respectively, and numbers denote different charge states. Molecular masses of the different protein forms (averaged over all detected charge states) are also presented.

The oligomeric state of zebavidin was analysed by native mass spectrometry (native-MS) analysis. Mass spectra obtained under native conditions and at low ionic strength (10 mM ammonium acetate, pH 7) at a protein (monomer) concentration of 10 μM without biotin (Figure 3A) revealed the presence of both protein monomers (charge states 7+ and 8+) and dimers (charge states 9+ to 11+) in solution. The determined mass of the dimer (27,177.34 ± 0.01) proves its noncovalent character without any ligands attached. In addition, very small peaks at ~*m*/*z* 3500-4000 were also detected corresponding to the protein tetramer. In contrast, in the presence of biotin only the protein tetramer (54353.3 ± 0.4 Da; charge states 14+ to 16+) was detected (Figure 3B); addition of a 3-fold molar excess of biotin increased the tetramer mass by ~980 Da consistent with the saturative binding of four biotin molecules. We also tested whether the protein tetramers could be stabilized at a higher ionic strength. Indeed, a mass spectrum acquired in 500 mM ammonium acetate, in the absence and presence of biotin (Figure 3C-D), revealed only the presence of protein tetramer. No protein monomers or dimers were detected under these conditions. The dynamic oligomeric state of zebavidin was further confirmed by analytical gel filtration in two different buffer systems and three different NaCl concentrations (0 mM, 100 mM, 650 mM). In a sodium phosphate buffer (50 mM NaH_2_PO_4_/Na_2_HPO_4_, 2 mM EDTA, pH 7) the protein eluted in a single sharp peak, independent of the salt concentration or the presence of biotin (Figure S3). The determined molecular masses (38.9-47.3 kDa) (see also Table S2) were comparable to that of bacterially expressed chicken avidin, which displayed a size of 39.5 and 38.2 kDa in the absence and presence of biotin, respectively. The determined masses were thus smaller than the theoretical masses of the tetramers. This behaviour has also been observed for the other bacterially expressed avidins, including biotin-binding protein A (BBP-A) [27] and avidin-related protein 4 (AVR4) [33], all of which have an apparent molecular mass of approximately 40 kDa in gel filtration analysis. This behaviour is believed to be caused by column interaction due to a high isoelectric point of the proteins (pI > 7.5). This is supported by AVR2, an avidin-related protein with an isoelectric point of 4.7, which displays the molecular mass of 55.1 kDa in gel filtration analysis, which is close to the value as determined by MS analysis (57.2 kDa) [33]. For conditions without sodium chloride, the elution peak was delayed by 2 ml. This delay was found not to be due to a difference in the size of the protein, but rather due to the differences in the performance of the column; low ionic strength of the mobile phase also affected the elution of protein standard accordingly (Figure S4). Similar elution volumes were observed in ammonium acetate buffer (10 mM ammonium acetate, 0.05% β-mercaptoethanol, pH7) at salt concentrations of 100 and 650 mM (Figure S3 A&B). In the absence of salt, it was not possible to obtain an elution peak in the absence of biotin (Figure S3A). In the presence of biotin the peak was not sharp, displaying a maximum at 17.6 ml and a shoulder towards higher elution volumes (Figure S3B).

 We also used an SDS-PAGE-based analysis to determine the oligomeric assembly of zebavidin at different temperatures. In the absence of biotin, the protein migrated as a monomer, even at ambient temperatures. In the presence of biotin, at ambient temperatures, the protein did not migrate as a perfect band, but it instead migrated in a smeared band indicating that the oligomer disassembled within the timescale of the analysis. The smeared band was visible up to a temperature between 60 °C and 70 °C, after which only monomeric species were observed (Figure S5). In comparison, chicken avidin exists as a clear tetramer up to 60 °C in the absence of biotin and up to 90 °C in the presence of biotin in this assay [55].

### Thermal stability

**Figure 4:**
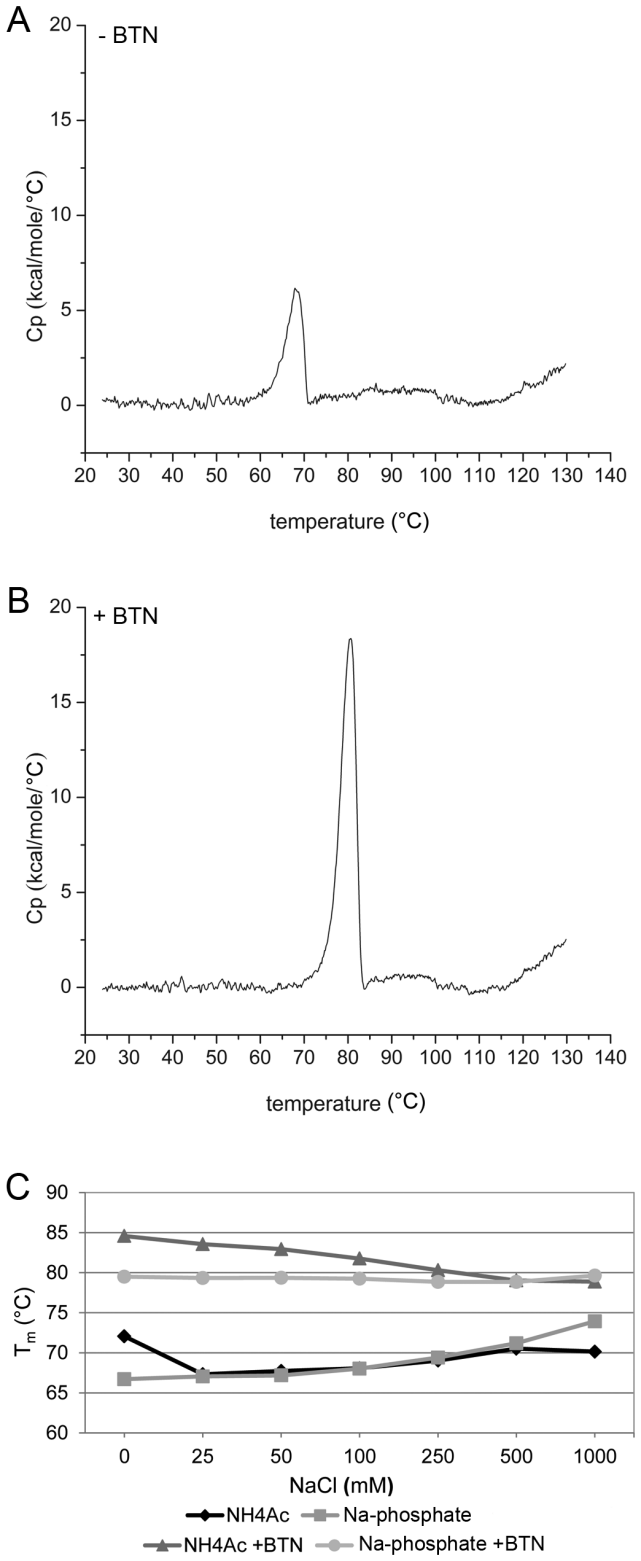
Thermal stability of zebavidin. DSC thermogram of 30 µM zebavidin in the absence (A) and in the presence (B) of 90 µM biotin (BTN). Protein was analysed in 50 mM Na_2_HPO_4_/NaH_2_PO_4_, 100 mM NaCl, 2 mM EDTA, pH 7. (C) Thermal transition midpoints of zebavidin as a function of the sodium chloride concentration in ammonium acetate (NH_4_Ac) or sodium phosphate buffer (Na-phosphate).

The thermal stability of zebavidin was analysed by DSC. The thermal transition midpoint (T_m_) in the absence and presence of biotin were 67.8 °C and 80.0 °C, respectively (Figure 4A&B). These values are considerably lower than those measured previously for chicken avidin (83.5 °C and 117.0 °C (+biotin)) [56] and for bacterial streptavidin (75.0 °C and 112.0 °C (+biotin)) [57]. The moderate increase in T_m_ (12.2 °C) in the presence of biotin also suggests a lower binding affinity than that observed for avidin, which is dramatically stabilised by the addition of biotin (ΔT_m_ = 41.8 °C). In comparison, ΔT_m_ = 21.2 °C have been measured for AVR2 in a DSC analysis after the addition of biotin [33]. According to our knowledge, zebavidin has the lowest thermal stability of the “natural” avidins characterised to date. Another avidin with a substantially reduced thermal stability is BBP-A. This protein exhibited two peaks in the DSC analysis in the absence of biotin at 51 °C and 68 °C, whereas biotin increased the T_m_ value to 103.4 °C [27].

 In order to analyse the influence of ionic strength on the thermal stability, T_m_ values were determined at different sodium chloride concentrations in either sodium phosphate or ammonium acetate buffer. With both buffer systems, clear trends could be observed with increasing salt concentrations (Figure 4C, see also Table S3 and Figure S6). In the absence of biotin, the thermal stability increased slightly with increasing salt concentration in both buffer systems. Surprisingly, in the presence of biotin the thermal stability decreased with increasing salt concentrations in ammonium acetate buffer, whereas the thermal stability stayed constant in the sodium phosphate buffer.

### Zebavidin biotin binding

**Figure 5:**
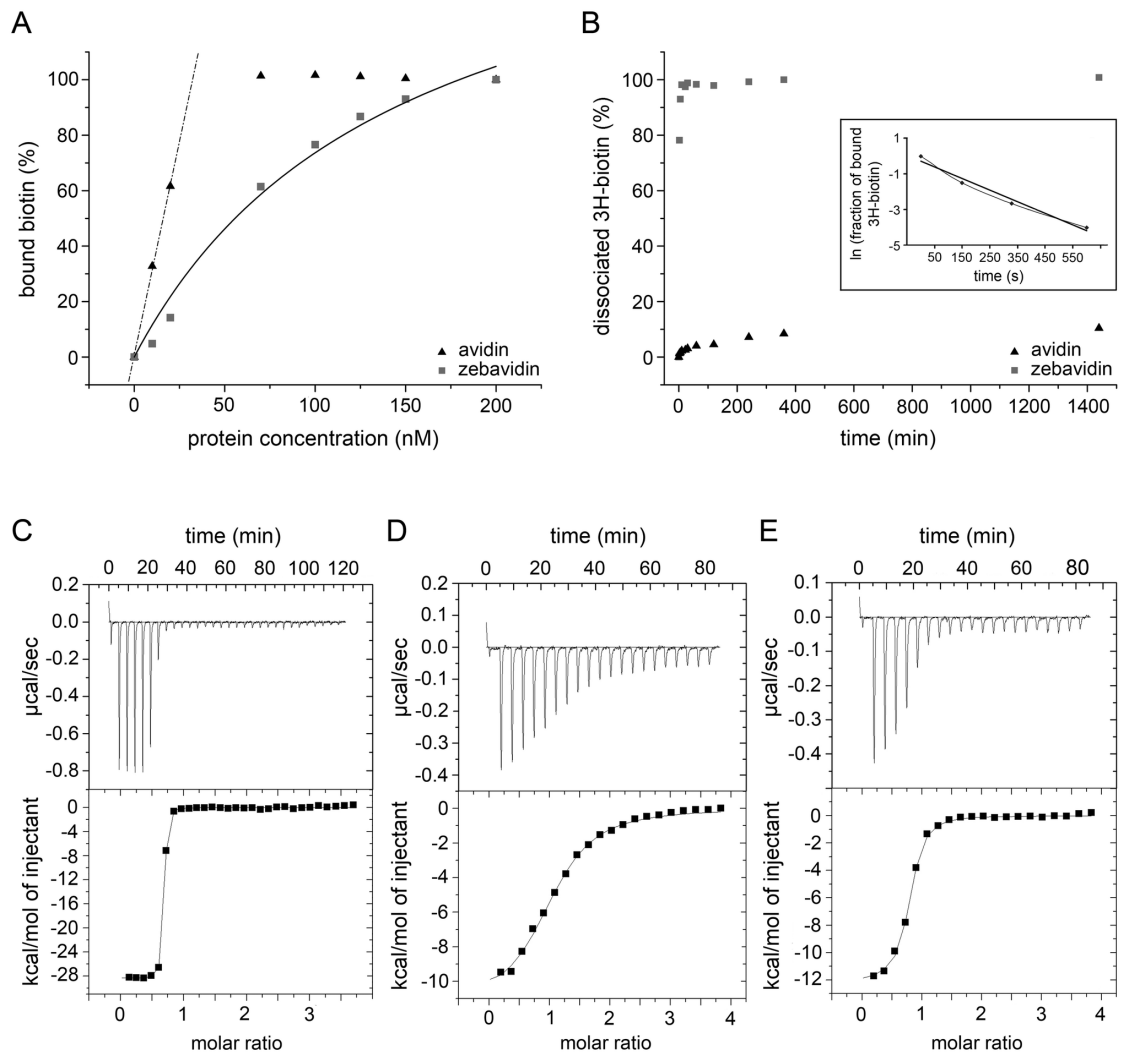
Biotin binding of zebavidin. (A) Determination of the binding affinity to fluorescently labelled biotin. The amount of bound ligand was determined by following the quenching of the fluorescence of the probe. A non-linear regression has been used to fit a binding curve to the zebavidin data. Avidin showed a very high affinity to the probe, and the line has been fitted to the first data points in order to visualise the tight binding at lower protein concentrations. (B) The dissociation of radio-labelled [^3^H]-biotin from the protein-ligand complex after addition of cold biotin measured at different time points. The inset shows the fit for the first data points of zebavidin used for the determination of the dissociation rate constant. (C) ITC binding thermogram of ligand titration to zebavidin. (D) Desthiobiotin titration to zebavidin. (E) Biotin titration to zebavidin-desthiobiotin. (C-E) Top panel: raw ITC data. (C-E) Bottom panel: binding isotherm derived from integrated heats.

In fluorescence spectroscopy, affinity for a fluorescent biotin conjugate was determined by titration of the protein into the dye. The equilibrium dissociation constant (K_d_) between zebavidin and the fluorescent biotin conjugate ArcDia BF560 was 1.4 · 10^-7^ M. In comparison, the affinity of avidin to the probe was too high to be determined by this method (Figure 5A). Furthermore, zebavidin showed rapid and complete dissociation of the fluorescent biotin conjugate ArcDia BF560 at 25 and 50 °C after the addition of free biotin (>90% of the complex dissociated within less than ten seconds, suggesting a k_diss_ value in the range of 0.1 - 1 s^-1^, data not shown). It was therefore not possible to determine the dissociation rate constant accurately using this method. In contrast, the dissociation rate constant of chicken avidin to the fluorescent biotin conjugate has been determined to be 2.3 · 10^-5^ s^-1^ at 25 °C [20].

 In the radioactive biotin ([^3^H]biotin) dissociation study, a dissociation rate constant k_diss_ of 6.5 · 10^-3^ s^-1^ was measured for zebavidin. This is ~100,000-fold higher than the dissociation rate of [^3^H]biotin determined for chicken avidin by extrapolation from measurements performed at higher temperatures using a global fit model (5.0 · 10^-8^ s^-1^) [33]. An indication of the reduced affinity of zebavidin for [^3^H]biotin relative to the affinity of other avidins was also found when the assay was initialised: the amount of free ligand immediately prior to the addition of cold biotin was 28% in the zebavidin assay and ~3% in the avidin assay. In addition, the majority of [^3^H]biotin was released from zebavidin within five minutes after the addition of a 1000-molar excess of cold biotin, and the release reached 100% within six hours (Figure 5B). For avidin, the dissociation of [^3^H]biotin was comparably lower, with only 10% of the probe being released 24 hours after addition of a 1000-molar excess of cold biotin.

 The dissociation rate of conjugated biotins from avidins is faster because the conjugate prevents interactions between the residues of the L3,4-loop and biotin. The structural details behind this phenomenon have been discussed in previous publications [58,59].

 Isothermal titration calorimetry analysis of biotin binding to zebavidin showed an affinity constant in the nanomolar range, which lies at the sensitivity limit of the instrument and therefore resulting in rather high error values of over 35% (Figure 5C and Table 2). Because of the low reliability of ITC for quantification of extremely tight binding, a competitive binding experiment was performed using desthiobiotin. This analysis gave a similar affinity constant, but with substantially decreased deviation in between two measurements (Figure 5D&E and Table 2).

**Table 2:** Biotin-binding constants and enthalpies determined by ITC at 40 °C.

**protein**	**ligand**	K_a_	**K_d_**	**ΔH**	**ΔS**	**ΔG**
		(x10^7^ M^-1^)	(x10^-9^ M)	(kcal/mol)	(cal/molK)	(kcal/mol)
zebavidin	biotin	19.5/42.3	5.13/2.36	-26.3/-28.3	-46.1/-51.0	-11.9/-12.4
zebavidin	desthiobiotin	0.15/0.14	660/700	-11.1/-11.3	-7.1/-8.0	-8.9/-8.8
zebavidin- desthiobiotin	biotin	20.8/22.6	4.8/4.4	-22.9/-22.6	-38.1/-38.3	-11.9/-12.0

Values from two independent measurements are displayed.

### X-ray structure of zebavidin

**Figure 6:**
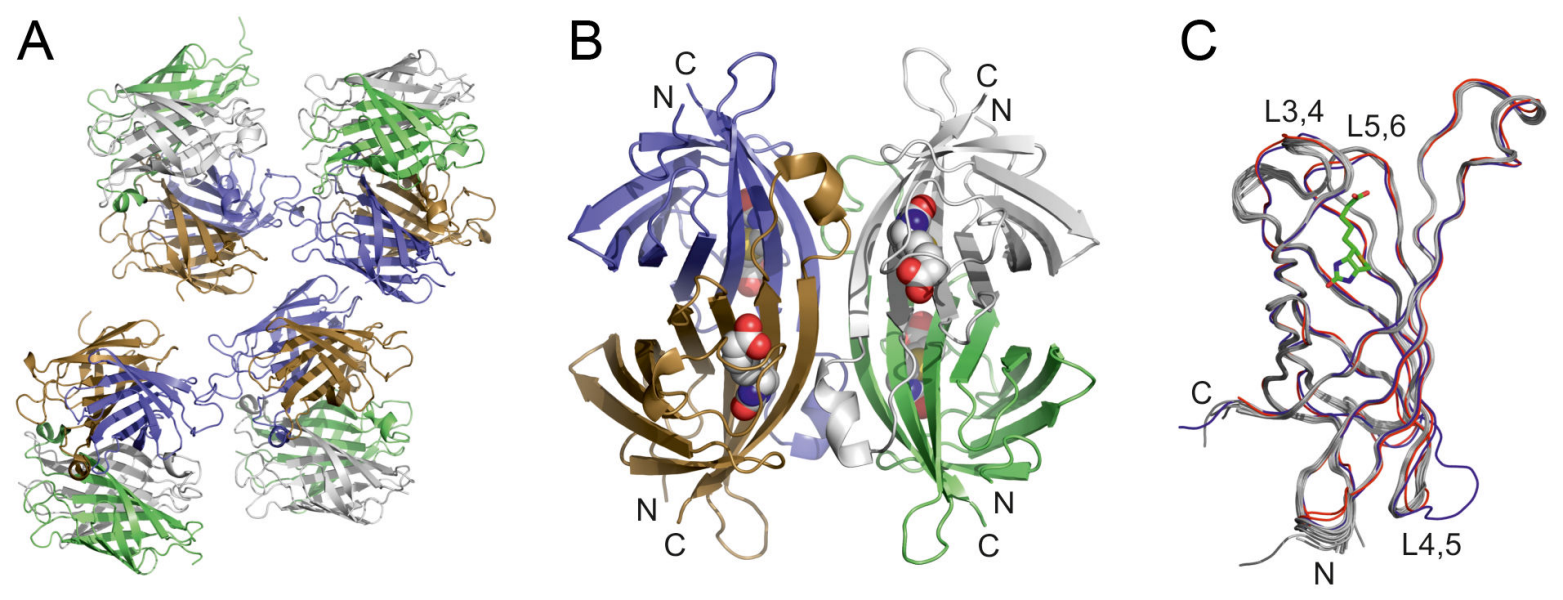
X-ray structure of zebavidin. A cartoon model of the contents of the asymmetric unit (A) and a tetramer (B) are coloured as follows: subunit I, blue; subunit II, green; subunit III light grey; and subunit IV, brown. (C) Ribbon representation of superimposed chains A-P of zebavidin (light grey), chain A of chicken avidin (blue) [PDB:1AVD] and chain A of xenavidin (red) [PDB:2UYW]. The biotin molecules of zebavidin are shown as space-filling models (B) or as a stick model (C). The N-termini (N) and C-termini (C) are indicated (B-C), as well as the L3,4-loop, L4,5-loop and L5,6-loop (C).

**Figure 7:**
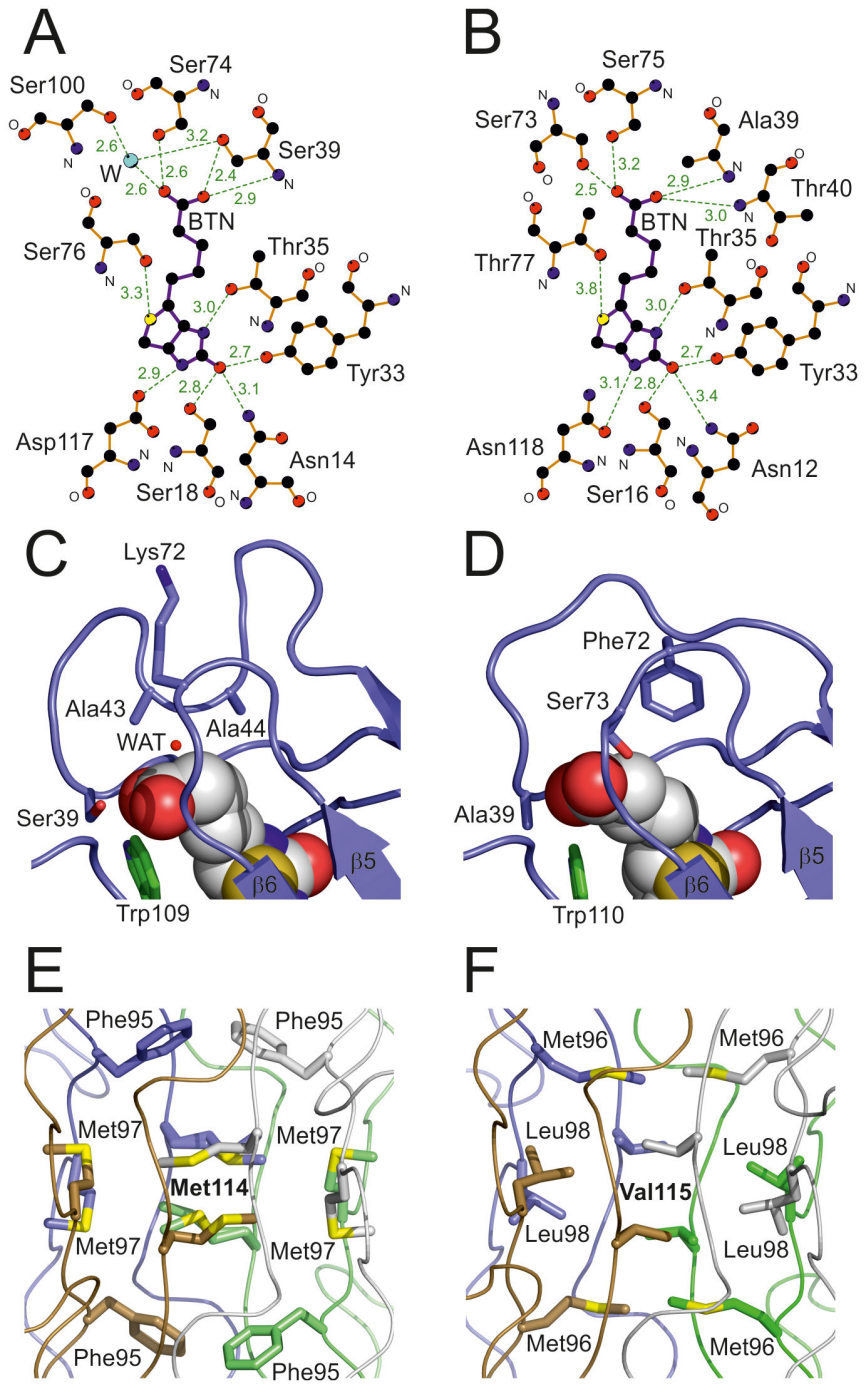
Unique structural properties of zebavidin. (A-B) A diagram showing hydrogen bonds to biotin (BTN) in zebavidin (A) and chicken avidin [PDB:1AVD] (B). Nitrogen atoms are coloured blue, oxygen atoms red, carbon atoms black and sulphur atoms yellow. A structural water molecule (W) is shown in cyan. The hydrogen bonds are drawn as dashed green lines and their distances shown in ångströms. The main-chain nitrogen (N) and oxygen (O) atoms are labelled. (C-D) Side chains of selected residues lining the valeric acid moiety of biotin in (C) zebavidin and (D) chicken avidin [PDB:1AVD] are shown as blue stick models, the oxygen and nitrogen atoms are coloured red and dark blue, respectively. The side chain of a tryptophan residue from a neighbouring subunit is coloured green. A structural water molecule (WAT) is indicated by a small red sphere (C). Biotin molecules are shown as space-filling models and the β5 and β6 strands are labelled. (E-F) The tetramer core of zebavidin (E) and chicken avidin (F) is shown as ribbon models. The subunits I-IV are coloured as in Figure 6A&B. Side-chain atoms of selected residues are shown as stick models; sulphur atoms are indicated by yellow colour.

Zebavidin crystallized in the space group *P*2_1_2_1_2 and, surprisingly, contained 16 monomers (four homotetramers) in the asymmetric unit (Figure 6A; see Table 1 for structure determination statistics). The homotetramer of zebavidin (Figure 6B) resembles that of chicken avidin and is a typical “dimer of dimers”. Each of the 16 monomers in the asymmetric unit is structurally highly similar to each other (Figure 6C) and the Cα atoms of the monomers superimpose with a root mean square deviation (rmsd) of less than 0.3 Å; the tetramers superimpose with the same accuracy. When compared to other eukaryotic avidin structures, chicken avidin [PDB: 1AVD, 2AVI] [52,60] and xenavidin [PDB: 2UYW] [10], the rmsd (Cα-atom positions) is 0.7 Å and 0.6 Å, respectively. The sequence identity between zebavidin and chicken avidin and xenavidin is less than 40%. The major structural differences are found at the biotin-binding loop, the L3,4-loop, which is three or more residues longer in zebavidin than in the other known avidin structures; the sequence and conformation of the L3,4-loop is also unique and especially affects the conformation of the adjacent L5,6-loop, which, in turn, is one amino acid shorter than in other avidin structures (Figure 6C, Figure 7A-D). When compared to its closest homolog, chicken avidin, there are clear differences in the L4,5-loop of zebavidin, too.

 The unique L3,4-loop and L5,6-loop affect the mode of biotin binding in zebavidin (Figure 7A), even though most biotin-binding residues are conserved in zebavidin when compared to other avidins. Highly conserved residues include Asn14, Leu16, Ser18, Tyr33, Thr35, Val37, Ser76, Trp78, Trp96, Trp109 (from another subunit), and Asp117 that surround the bicyclic ring moiety (ureido/tetrahydroimidizalone ring fused with a tetrahydrothiophene ring) of biotin; they also have very similar conformations as the equivalent residues in the structures of chicken avidin [PDB: 1AVD, 2AVI] and xenavidin [PDB: 2UYW]. Even though Asp117 is replaced in avidin/xenavidin by Asn118/Asn117 and Ser76 by Thr77/Thr76, and Trp78 by Phe79 in avidin, these substitutions are common within the avidins. In contrast, Glu38, Ser39, Thr40, Ala43, Ala44, Trp70, Ser74 and Leu98, which surround the valeric acid moiety of biotin, mostly have unique interactions (Figure 1B; Figure 7A-D). For example, the side-chain oxygen atom of Ser39 of zebavidin may form a hydrogen bond with one oxygen atom of the valeric acid moiety of biotin (or, depending on the chain, with a side-chain nitrogen atom of Arg113); in avidin/xenavidin a hydrophobic residue Ala39/Leu41 is found. The Cα-atoms of Ala43 and Ala44 (L3,4-loop) point towards the carbon atoms of the valeric acid moiety, Ala44 occupying the space that is occupied by the side chain of Phe72/Phe71 of the L5,6-loop in avidin/xenavidin (Figure 7C&D). The L5,6-loop is shorter than in other avidins and a residue equivalent to Phe72 in avidin is missing, affecting the positioning and conformation of Lys72, which is not in direct contact with biotin even though the equivalent residue Ser73/Ser72 in avidin/xenavidin is hydrogen bonded to biotin. However, in zebavidin a structural water molecule is found at a position matching that of the side-chain oxygen atom of Ser73 of avidin suggesting that this water molecule, and at least two other structural water molecules near the valeric acid moiety, may be important for biotin binding (Figure 7A&C).

 The subunit interfaces of zebavidin have many interactions unique within avidin proteins, the most characteristic ones being located at the core of all subunits (Figure 7E&F). The electron density maps indicates high thermal motion and the presence of alternative rotamers for several out of the sixteen Met114 residues found in the cores of four tetramers of the asymmetric unit. The equivalent residue in avidin/xenavidin is Val115/Val114. Moreover, Phe95 (weak electron density for many of the side chain atoms) and Met97 are located near the “Met114-core”. Together with Met114 and Glu116, they are the key residues at the subunit 1-2 and 1-3 interfaces (IF1,2 and IF1,3 interfaces; subunit numbering according to Livnah et al, 1993 {{17 Livnah,O. 1993;}}); these residues are also unique to zebavidin. The residues equivalent to Phe95, Met97 and Glu116 in zebavidin are Met96/Met95, Leu98/Leu97 and Ile117/Ala116 in avidin/xenavidin. To our knowledge, Phe95, Met97, Met114 and Glu116 are not found in any other characterized members of the avidin protein family, suggesting that the architecture of the IF1,2 and IF1,3 interfaces of zebavidin are at least partially responsible for the observed physicochemical properties (see above), such as low thermal stability.

 In addition to shared interactions with the IF1,2 and IF1,3 interfaces, the large IF1,4 interface of zebavidin is defined with many additional non-conserved interactions, too; including the salt bridge formed by residues Glu28 and Arg30, and numerous hydrophobic and weak interactions in which residues Ile53, Met55, Ser57, Thr63, Ser65, Ser67, Leu69, Ala77, Val79, Lys93, Arg99, Leu105 and Thr112 are involved. Probably the most unique feature of the IF1,4 interface is the presence of a free cysteine residue, Cys75. This cysteine may form weak hydrogen bonds with the side chain oxygen atoms of Thr63 and Ser65, or with the main chain oxygen atom of Val64, but it is not close to any other cysteine residues. None of the known other avidin structures has a cysteine at an equivalent position.

## Discussion

The availability of genetic tools in zebrafish makes it an attractive model for studying the function of avidin. Although much effort has been put into investigating the biological role of avidin [61], a comprehensive understanding is still missing. The characterisation of zebavidin reported here provides new insights towards a more complete picture of the biological importance of avidins. 

 Besides avidin, which is present in egg white, chicken appears to have a set of different biotin-binding proteins localised in the egg yolk; these proteins have been reported to be responsible for the deposition of biotin into the egg yolk for the developing embryo [27,51,62,63]. The biotin-binding proteins are expressed in liver and show lower thermal stability and lower biotin-binding affinity in comparison to avidin [62,64]. In contrast to chicken, zebrafish appears to carry only one avidin-like gene in its genome, which brings up the question whether zebavidin fulfils the role of avidin or the biotin-binding protein. Like biotin-binding protein A [27], zebavidin has low thermal stability and lower biotin-binding affinity. However, the highest expression of zebavidin was observed in the gonads of female fish and no expression could be observed in the liver. This suggests that zebavidin could have a functional role similar to avidin, possibly functioning as an anti-microbial agent. It is possible that zebrafish is missing a biotin-binding protein that is responsible for the transport of biotin to the oocyte and that the transport of the vitamin happens through a different mechanism. However, not much is known so far about the transport and uptake of vitamins by the developing oocyte [65]. Our results indicate that zebavidin is not necessarily needed during the development of the embryo and might not be expressed during embryogenesis (Figure 2).

 Differences in the biotin-binding properties between zebavidin and the chicken avidin were observed already during the purification stage - it was not possible to purify zebavidin with 2-iminobiotin affinity chromatography. Avidin-like proteins with low or no affinity towards 2-iminobiotin have also been found previously, such as AVR2 having negligible affinity for 2-iminobiotin [33]. In contrast to avidin, which sticks almost irreversibly to the biotin-coated affinity resin, both zebavidin and AVR2 can be eluted from the resin with a relatively mild treatment. Moreover, the biotin complexes of zebavidin and AVR2 have similar dissociation rate constants at 22 °C: k_diss_ = 6.5 · 10^-3^ s^-1^ for zebavidin, and k_diss_ = 1.5 · 10^-4^ s^-1^ for AVR2, which are both substantially higher than that determined for chicken avidin (k_diss_ = 5.0 · 10^-8^ s^-1^) [33]. Structural differences in the L3,4- and L5,6-loop as well as the differences in the residues in contact with biotin, especially hydrogen bonding to the valeric acid moiety of biotin, are likely to have a cumulative impact on the observed lower biotin-binding affinity of zebavidin in comparison to other avidins. The observed oligomeric instability (see below) may contribute to the decrease in the biotin-binding affinity, too.

 Most of the avidins analysed to date form tetramers in solution. Our native-MS and gel filtration analysis indicated that zebavidin forms stable tetramers in solutions having an appropriate ionic strength, but dissociates into monomers and dimers in the low ionic strength. The addition of biotin also stabilised the tetrameric assembly and only the tetrameric form was detected in the presence of biotin. Such transient oligomerization has not been described for other natural avidins. For example, rhizavidin was found to be exclusively dimeric, both in the absence and presence of biotin [13] and also at a lower ionic strength (e.g. in 10 mM ammonium acetate buffer). Bradavidin II has been found to form oligomers as a function of protein concentration, but no clearly defined oligomeric states were observed and biotin had no effect on the oligomeric state [66]. However, transient oligomerisation was observed for mutant avidins. Monomeric streptavidin mutants containing either mutation Ser45Ala or Asp128Ala were stable monomers in the absence of biotin, but in the presence of biotin, they partially formed tetramers [67]. Similarly, a monomeric recombinant avidin mutant with mutations Asn54Ala, Asn69Ala, Met96Ala, Val115Ala and Ile117Ala was tetrameric in the presence of biotin [68]. At the structural level, the Met114 residues forming the core of all tetramers in the crystal structure of zebavidin, together with the IF1,2 interface residue Phe95 and the IF1,3 interface residue Met97, may be responsible for the transient oligomeric behaviour that we observed experimentally. The methionine side-chain is long, unbranched and inherently very flexible. The four Met114 and four Phe95 residues at the core of the tetramer showed high thermal motion, which may lead to instability of the zebavidin oligomers. The free and relatively polar Cys75 in nonpolar environment at the IF1,4 interface of zebavidin, together with several other unique interactions found at the same interface, may also weaken the quaternary structure, enabling transient oligomeric states under different buffer conditions.

 The observed thermal stability of zebavidin was lower than that observed for other avidins. Like the transient oligomeric stability, the low thermal stability can also be explained by the unique subunit architecture of zebavidin, as described above. In addition, the dimer-dimer interface contains two hydrogen-bonded pairs of negatively charged glutamic acid residues (Glu116), which we propose would contribute to the stability of the zebavidin tetramers. Even though this site is one of the key interactions sites at the IF1,3 subunit interface of all tetrameric avidins characterized to date, it is not well conserved. Interestingly, mutation of the corresponding site in streptavidin from histidine to a negatively charged aspartic acid (His127Asp) disrupted the dimer-dimer interface [69]. Other previously reported mutagenesis studies have also shown that the subunit interfaces are important for the stability of the avidin. For example, chicken avidin was stabilised by the Ile117Tyr mutation, which resulted in a more compact packing of the side chains at the IF1,3 interface [70]. Analogously, introducing a disulphide bond between subunits 1 and 3 by the mutation Ile117Cys led to increased thermal stability, too [56]. In contrast, the IF1,2 interface mutant Met96His substantially destabilised the avidin tetramer [71]. 

 The low thermal stability of zebavidin might reflect the temperature of the natural living environment of zebrafish. Whereas chicken has a body temperature of approximately 40 °C, zebrafish lives at a relatively constant temperature of approximately 25 °C. The eggs of chicken and zebrafish may experience even more deviations in terms of the temperature. Therefore, one could speculate that zebrafish avidin does not need to be as thermostable as chicken avidin in order to resist the changes in its external environment.

## Conclusion

Zebavidin is an avidin-like protein from zebrafish and highly expressed in the gonads of adult fish but our preliminary results suggest that it is not critical for the development of the embryo. Our results do not exclude a potential role for zebavidin as an anti-microbial agent as suggested for chicken avidin [5,6,72], and additional experiments with e.g. bacterial infected embryos and adult zebrafish would be needed to fully elucidate the physiological role of zebavidin. Structural and functional characterisation confirmed the hypothesis that zebavidin is a new member of the avidin protein family and binds tightly to biotin, even though the biotin-binding affinity of zebavidin is substantially lower than that measured for avidin or streptavidin. Moreover, the oligomeric state of zebavidin seems to be unstable at least in conditions with low ionic strength, which could be explained by the unique subunit interface architecture of the protein.

## Supporting Information

Figure S1 **Isolation of zebavidin from zebavidin oocytes.** SDS-PAGE of fractions taken during protein isolation with biotin sepharose. Oocytes from five different individuals were analysed (1-5). R1: biotin sepharose incubated with wash fraction before homogenisation of oocytes; R2: biotin sepharose incubated with clarified homogenised oocytes after one wash; W3: wash fraction of R2; FT1: non- bound proteins from R1; FT2: non- bound proteins from R2. Protein bands A-D, which were analysed by LC-MS/MS, are indicated by a black rectangle. Selected bands A and B came from samples taken during the isolation process from the same sample of oocytes. Selected bands C and D resulted from another sample of oocytes. For band A and D, biotin sepharose was incubated with the wash fraction before sonication of the oocytes and represent biotin-binding protein that is located in the oviduct of the mother. For band B, biotin sepharose was incubated with the ruptured oocytes and represent biotin-binding protein that is located inside the oocyte. Band C was taken from the supernatant fraction after incubation of wash fraction with biotin sepharose and represents non biotin-binding proteins. Zebavidin has two potential glycosylation sites at position 22 and 35. The reason for the observed unsharp zebavidin bands might therefore partly be due to heterogeneous glycosylation.
(TIF)

Figure S2 **Mass spectrometry analysis of zebavidin.** ESI FT-ICR mass spectra of 10 μM zebavidin in denaturing solution conditions (acetonitrile/water/acetic acid 49.5:49.5:1, v/v, pH 3.2).
(TIF)

Figure S3 **Analytical gel filtration elution diagrams of zebavidin in different buffer systems and salt concentrations.** Elution chromatograms in 10 mM ammonium acetate, pH 7 (NH_4_Ac) with 0, 100 or 650 mM NaCl in the absence (**A**) and presence (**B**) of biotin (BTN) and in 50 mM Na_2_HPO_4_/NaH_2_PO_4_, pH 7 (Na-phosphate) with different salt concentrations in the absence (**C**) and presence (**D**) of biotin.
(TIF)

Figure S4 **Analytical gel filtration elution diagrams of standard proteins in different conditions.** (**A**) In 10 mM ammonium acetate, pH 7 with 0, 100, 650 mM NaCl (NH_4_Ac). (**B**) In 50 mM Na_2_HPO_4_/NaH_2_PO_4_, pH 7 with 0, 100, 650 mM NaCl (Na-phosphate).
(TIF)

Figure S5 **Oligomeric state of zebavidin in dependence of temperature.** SDS-PAGE of chemically acetylated zebavidin incubated at variant temperatures in the absence and presence of biotin. M: molecular weight marker (kDa).
(TIF)

Figure S6 **Thermal stability of zebavidin in dependence of sodium chloride concentration.** (**A**) in 10 mM ammonium acetate buffer (NH_4_Ac) in the absence of biotin (-BTN). (**B**) in the presence of biotin (+BTN). (**C**) in 50 mM Na_2_HPO_4_/NaH_2_PO_4_ buffer (Na-phosphate) in the absence of biotin. (**D**) in the presence of biotin. Zebavidin concentration of 20 µM and biotin concentration of 60µM was used in all measurements.
(TIF)

Table S1 **Peptides from LC-MS/MS analysis matching zebavidin sequence.**
(DOCX)

Table S2 **Elution volumes and molecular weights (MW) of zebavidin in different conditions obtained by analytical gel filtration.**
(DOCX)

Table S3 **Thermal stability of zebavidin as a function of sodium chloride concentration obtained by DSC.**
(DOCX)
