# Supplementary figures and images for: Zebavidin - An Avidin-Like Protein from Zebrafish
Source: PLoS One. 2013 Oct 24;8(10):e77207. doi: 10.1371/journal.pone.0077207 (PMC3811995; doi:10.1371/journal.pone.0077207)

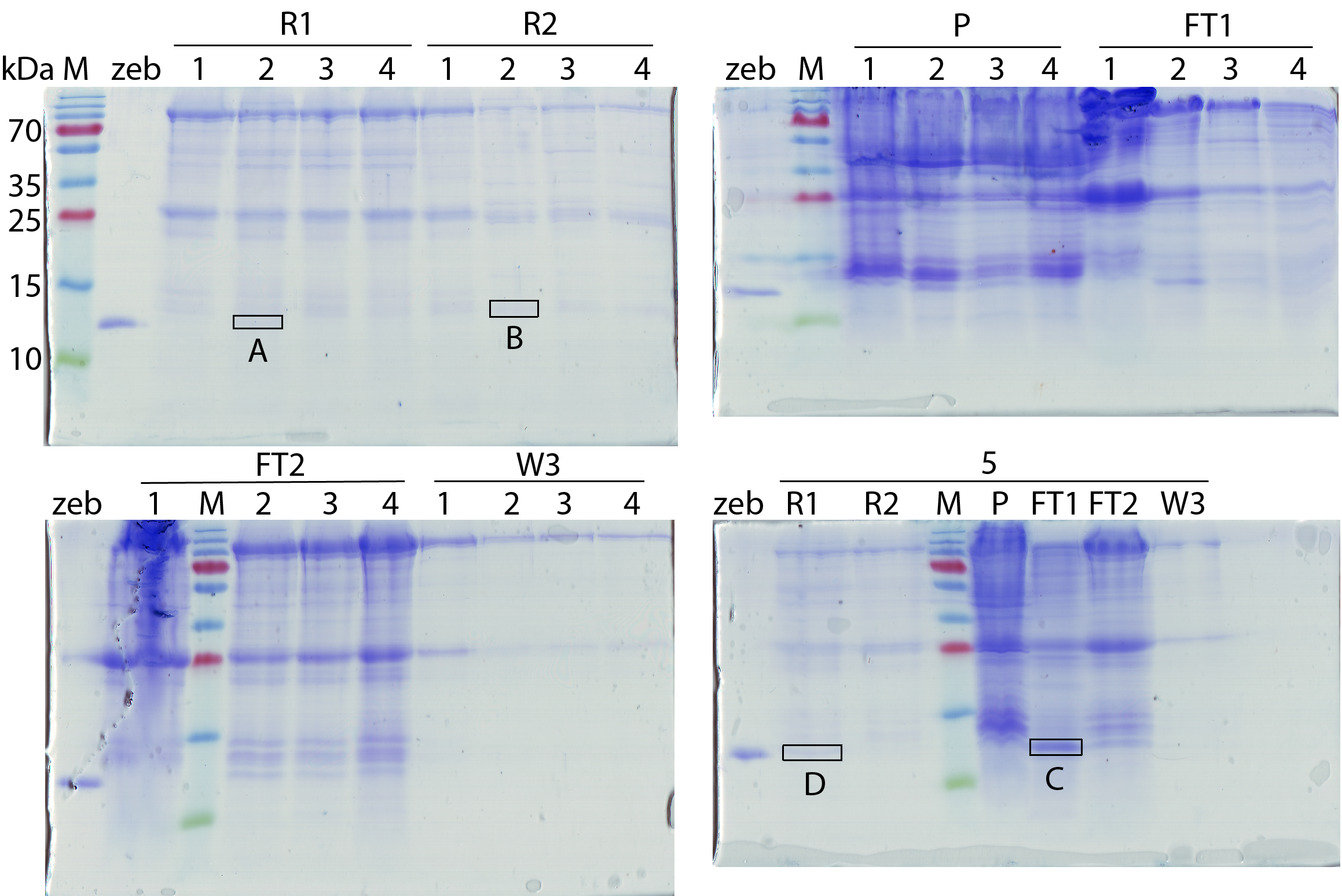

Supplement: Figure S1 — Isolation of zebavidin from zebavidin oocytes. SDS-PAGE of fractions taken during protein isolation with biotin sepharose. Oocytes from five different individuals were analysed (1-5). R1: biotin sepharose incubated with wash fraction before homogenisation of oocytes; R2: biotin sepharose incubated with clarified homogenised oocytes after one wash; W3: wash fraction of R2; FT1: non- bound proteins from R1; FT2: non- bound proteins from R2. Protein bands A-D, which were analysed by LC-MS/MS, are indicated by a black rectangle. Selected bands A and B came from samples taken during the isolation process from the same sample of oocytes. Selected bands C and D resulted from another sample of oocytes. For band A and D, biotin sepharose was incubated with the wash fraction before sonication of the oocytes and represent biotin-binding protein that is located in the oviduct of the mother. For band B, biotin sepharose was incubated with the ruptured oocytes and represent biotin-binding protein that is located inside the oocyte. Band C was taken from the supernatant fraction after incubation of wash fraction with biotin sepharose and represents non biotin-binding proteins. Zebavidin has two potential glycosylation sites at position 22 and 35. The reason for the observed unsharp zebavidin bands might therefore partly be due to heterogeneous glycosylation. (TIF) [file pone.0077207.s001.tif]

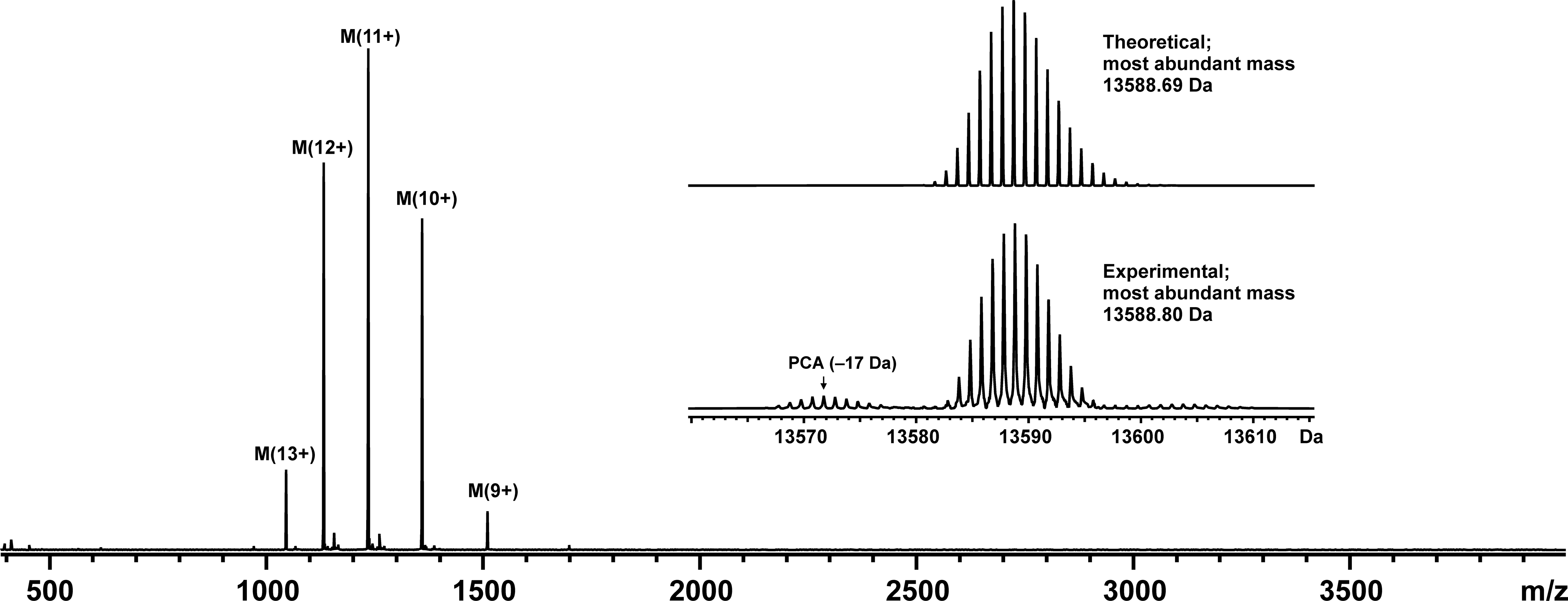

Supplement: Figure S2 — Mass spectrometry analysis of zebavidin. ESI FT-ICR mass spectra of 10 μM zebavidin in denaturing solution conditions (acetonitrile/water/acetic acid 49.5:49.5:1, v/v, pH 3.2). (TIF) [file pone.0077207.s002.tif]

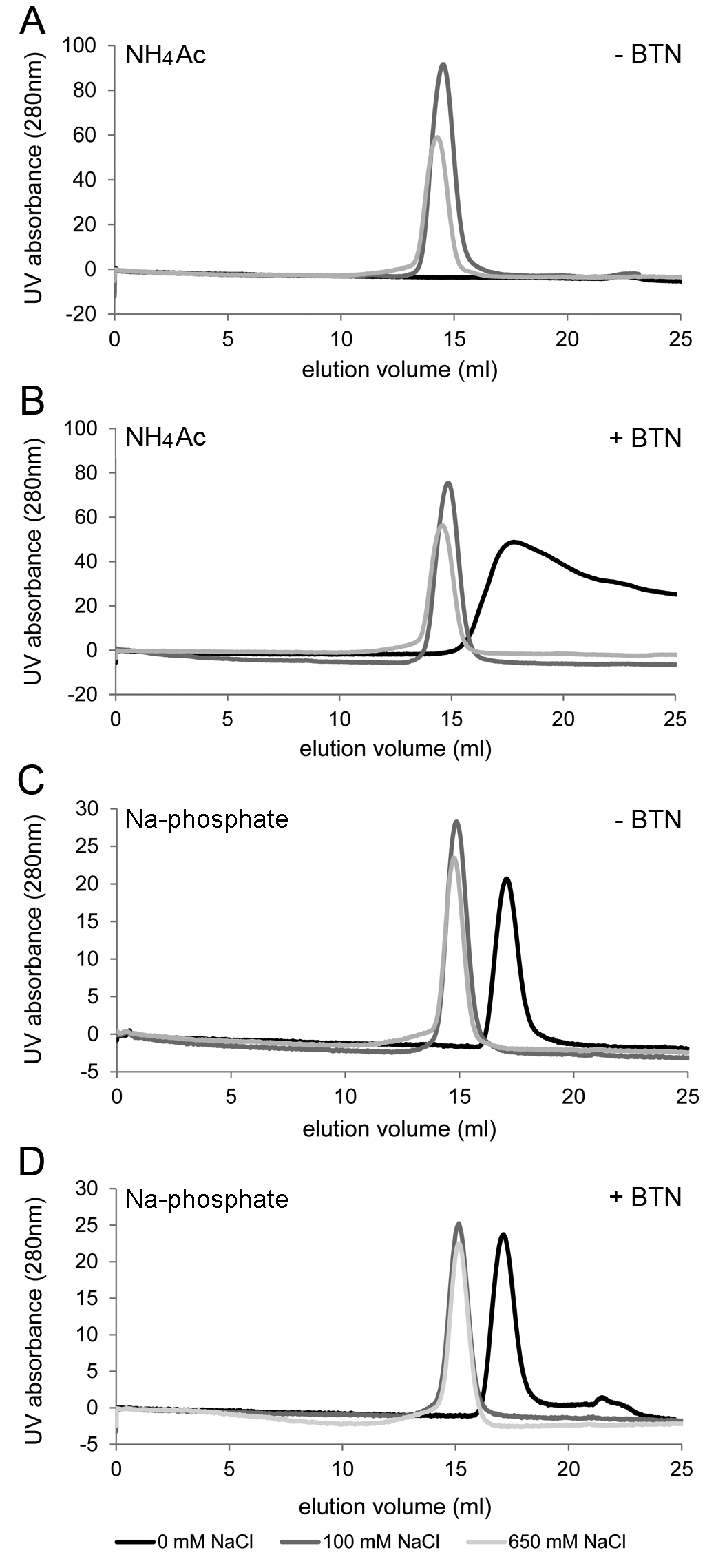

Supplement: Figure S3 — Analytical gel filtration elution diagrams of zebavidin in different buffer systems and salt concentrations. Elution chromatograms in 10 mM ammonium acetate, pH 7 (NH4Ac) with 0, 100 or 650 mM NaCl in the absence (A) and presence (B) of biotin (BTN) and in 50 mM Na2HPO4/NaH2PO4, pH 7 (Na-phosphate) with different salt concentrations in the absence (C) and presence (D) of biotin. (TIF) [file pone.0077207.s003.tif]

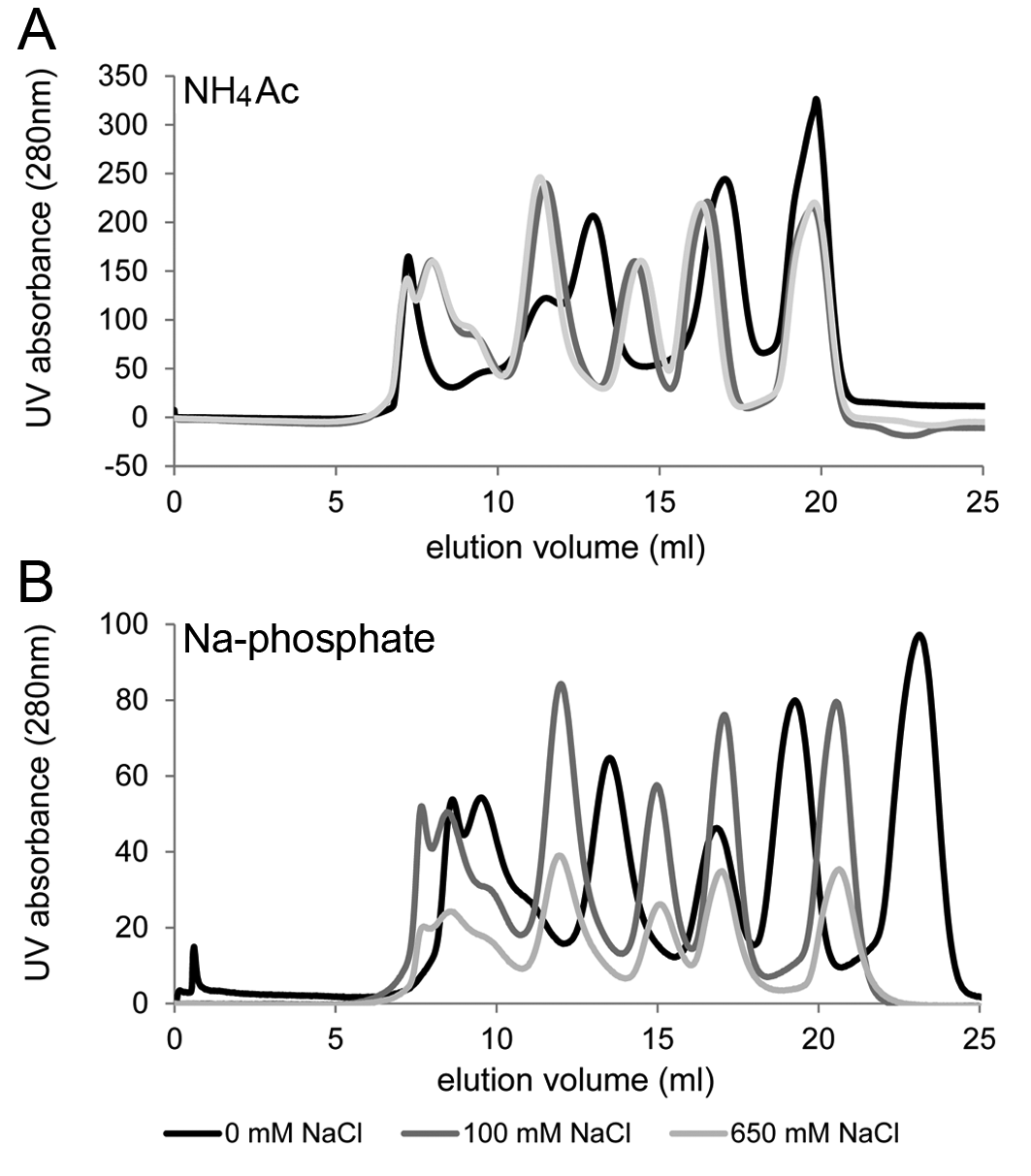

Supplement: Figure S4 — Analytical gel filtration elution diagrams of standard proteins in different conditions. (A) In 10 mM ammonium acetate, pH 7 with 0, 100, 650 mM NaCl (NH4Ac). (B) In 50 mM Na2HPO4/NaH2PO4, pH 7 with 0, 100, 650 mM NaCl (Na-phosphate). (TIF) [file pone.0077207.s004.tif]

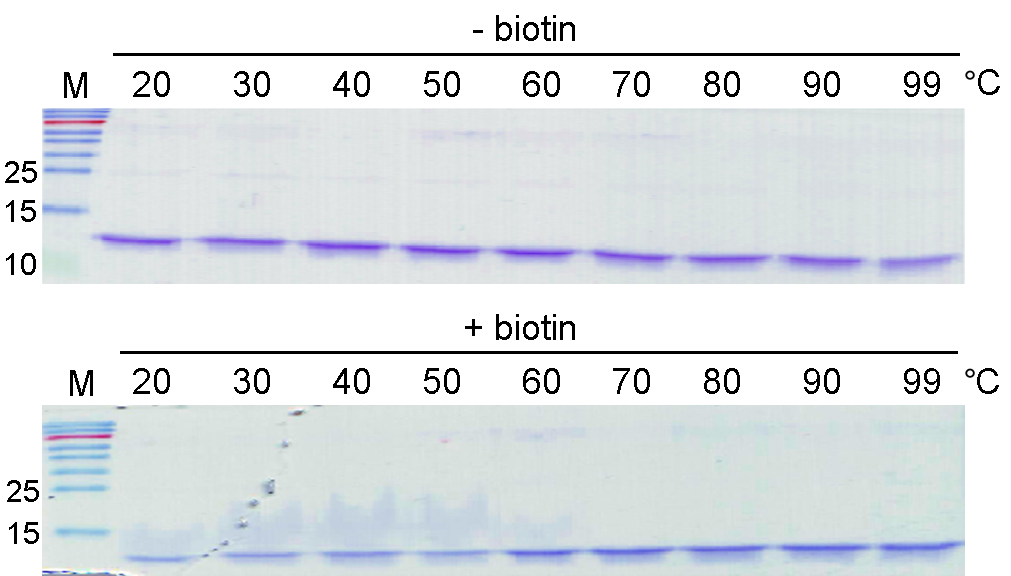

Supplement: Figure S5 — Oligomeric state of zebavidin in dependence of temperature. SDS-PAGE of chemically acetylated zebavidin incubated at variant temperatures in the absence and presence of biotin. M: molecular weight marker (kDa). (TIF) [file pone.0077207.s005.tif]

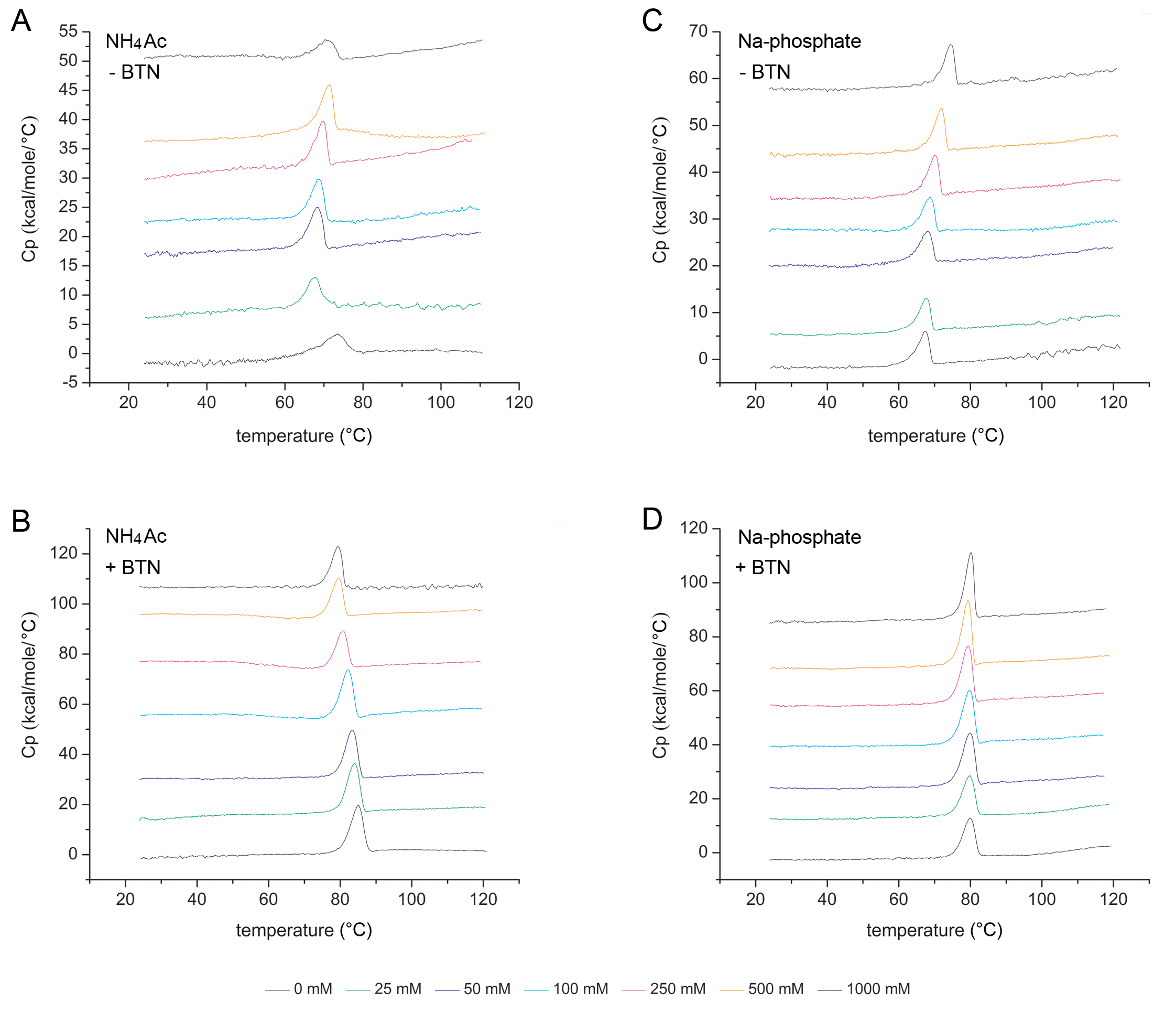

Supplement: Figure S6 — Thermal stability of zebavidin in dependence of sodium chloride concentration. (A) in 10 mM ammonium acetate buffer (NH4Ac) in the absence of biotin (-BTN). (B) in the presence of biotin (+BTN). (C) in 50 mM Na2HPO4/NaH2PO4 buffer (Na-phosphate) in the absence of biotin. (D) in the presence of biotin. Zebavidin concentration of 20 µM and biotin concentration of 60µM was used in all measurements. (TIF) [file pone.0077207.s006.tif]
