# Supplementary material for: Zebavidin - An Avidin-Like Protein from Zebrafish
Source: PLoS One. 2013 Oct 24;8(10):e77207. doi: 10.1371/journal.pone.0077207 (PMC3811995; doi:10.1371/journal.pone.0077207)
Supplement: Table S1 — Peptides from LC-MS/MS analysis matching zebavidin sequence. (DOCX) [file pone.0077207.s007.docx]

**Table S1** Peptides from LC-MS/MS analysis matching zebavidin sequence.

| **protein band** | **fraction** | **location** | **peptide match** | **peptide sequence** |
| --- | --- | --- | --- | --- |
| A | biotin sepharose bound | oviduct | 2 | SVADNLASAWGSTR |
|  |  |  |  | GVYQTAVESTR |
| B | biotin sepharose bound | oocyte | 4 | SVADNLASAWGSTR |
|  |  |  |  | GVYQTAVESTR |
|  |  |  |  | MGEDIFFK |
|  |  |  |  | TFWMLR |
| C | non-biotin binding |  | 0 |  |
| D | biotin sepharose bound | oviduct | 1 | SVADNLASAWGSTR |
