# Supplementary material for: Zebavidin - An Avidin-Like Protein from Zebrafish
Source: PLoS One. 2013 Oct 24;8(10):e77207. doi: 10.1371/journal.pone.0077207 (PMC3811995; doi:10.1371/journal.pone.0077207)
Supplement: Table S2 — Elution volumes and molecular weights (MW) of zebavidin in different conditions obtained by analytical gel filtration. (DOCX) [file pone.0077207.s008.docx]

**Table S2** Elution volumes and molecular weights (MW) of zebavidin in different conditions obtained by analytical gel filtration.

|  | **Sodium phosphate buffer** | | **Ammonium acetate buffer** | |
| --- | --- | --- | --- | --- |
| **Sample** | **Elution volume (ml)** | **MW (kDa)** | **Elution volume (ml)** | **MW (kDa)** |
| 0 mM NaCl |  |  |  |  |
| zebavidin | 17.1 | 40.0 | n.a. | n.a.* |
| zebavidin+ biotin | 17.1 | 38.9 | 17.6 | n.a.* |
| 100 mM NaCl |  |  |  |  |
| zebavidin | 14.9 | 44.2 | 14.5 | 39.9 |
| zebavidin+ biotin | 15.2 | 39.6 | 14.9 | 34.5 |
| 650 mM NaCl |  |  |  |  |
| zebavidin | 14.8 | 47.3 | 14.5 | 39.2 |
| zebavidin+ biotin | 14.9 | 44.8 | 14.6 | 38.0 |

* MW was not calculated due to disturbed elution of protein standard (see Supporting Figure S4)

n.a.: not available
