# Supplementary material for: Zebavidin - An Avidin-Like Protein from Zebrafish
Source: PLoS One. 2013 Oct 24;8(10):e77207. doi: 10.1371/journal.pone.0077207 (PMC3811995; doi:10.1371/journal.pone.0077207)
Supplement: Table S3 — Thermal stability of zebavidin as a function of sodium chloride concentration obtained by DSC. (DOCX) [file pone.0077207.s009.docx]

**Table S3** Thermal stability of zebavidin as a function of sodium chloride concentration obtained by DSC.

| **Buffer system** | **NaCl (mM)** | **T_m_ (°C)** | | **ΔT_m_ (°C)** |
| --- | --- | --- | --- | --- |
|  |  | **- BTN** | **+ BTN** |  |
| NH_4_Ac | 0 | 72.1 | 84.6 | 12.5 |
|  | 25 | 67.3 | 83.6 | 16.3 |
|  | 50 | 67.7 | 82.9 | 15.2 |
|  | 100 | 68.1 | 81.8 | 13.7 |
|  | 250 | 69.0 | 80.3 | 11.3 |
|  | 500 | 70.5 | 79.0 | 8.5 |
|  | 1000 | 70.2 | 78.9 | 8.7 |
| Na_2_HPO_4_/NaH_2_PO_4_ | 0 | 66.7 | 79.5 | 12.8 |
|  | 25 | 67.1 | 79.3 | 12.2 |
|  | 50 | 67.2 | 79.4 | 12.2 |
|  | 100 | 68.0 | 79.3 | 11.3 |
|  | 250 | 69.4 | 78.9 | 9.5 |
|  | 500 | 71.2 | 78.9 | 7.7 |
|  | 1000 | 73.9 | 79.6 | 5.7 |
